# Towards a comprehensive atlas of cortical connections in a primate brain: Mapping tracer injection studies of the common marmoset into a reference digital template

**DOI:** 10.1002/cne.24023

**Published:** 2016-06-03

**Authors:** Piotr Majka, Tristan A. Chaplin, Hsin‐Hao Yu, Alexander Tolpygo, Partha P. Mitra, Daniel K. Wójcik, Marcello G.P. Rosa

**Affiliations:** ^1^ Neuroscience Program, Biomedicine Discovery Institute Monash University Clayton VIC Australia; ^2^ Department of Physiology Monash University Clayton VIC Australia; ^3^ Nencki Institute of Experimental Biology Warsaw Poland; ^4^ Australian Research Council Centre of Excellence for Integrative Brain Function Monash University Node Clayton VIC Australia; ^5^ Monash Vision Group Monash University Clayton VIC Australia; ^6^ Cold Spring Harbor Laboratory Cold Spring Harbor New York USA

**Keywords:** digital atlas, marmoset, cerebral cortex, neuroanatomical tracing, image registration, Nissl staining, brain template

## Abstract

The marmoset is an emerging animal model for large‐scale attempts to understand primate brain connectivity, but achieving this aim requires the development and validation of procedures for normalization and integration of results from many neuroanatomical experiments. Here we describe a computational pipeline for coregistration of retrograde tracing data on connections of cortical areas into a 3D marmoset brain template, generated from Nissl‐stained sections. The procedure results in a series of spatial transformations that are applied to the coordinates of labeled neurons in the different cases, bringing them into common stereotaxic space. We applied this procedure to 17 injections, placed in the frontal lobe of nine marmosets as part of earlier studies. Visualizations of cortical patterns of connections revealed by these injections are supplied as Supplementary Materials. Comparison between the results of the automated and human‐based processing of these cases reveals that the centers of injection sites can be reconstructed, on average, to within 0.6 mm of coordinates estimated by an experienced neuroanatomist. Moreover, cell counts obtained in different areas by the automated approach are highly correlated (*r* = 0.83) with those obtained by an expert, who examined in detail histological sections for each individual. The present procedure enables comparison and visualization of large datasets, which in turn opens the way for integration and analysis of results from many animals. Its versatility, including applicability to archival materials, may reduce the number of additional experiments required to produce the first detailed cortical connectome of a primate brain. J. Comp. Neurol. 524:2161–2181, 2016. © 2016 The Authors The Journal of Comparative Neurology Published by Wiley Periodicals, Inc.

We present and validate a workflow for streamlined and reproducible mapping of the results of retrograde fluorescent tracer injections in the marmoset cerebral cortex into a common stereotaxic space. This proposed pipeline is a necessary methodological step towards the creation of a cellular‐level connectivity atlas of a primate brain, by allowing ready comparison of multiple cases, spatial‐based analyses, and future comparisons with other imaging modalities. The present focus is on the cerebral cortex, about which our laboratories have gathered a large amount of connectivity data over two decades. By comparing results derived through automated and classical (expert‐based) methods in the same cases, we also address important issues that are central to current attempts to generate comprehensive connectivity maps in complex brains, which are subdivided into many areas: the likely margin of error involved in assigning label to specific cortical areas due to the registration of multiple cases onto a common digital template, and the nature of the errors incurred.

Despite advances in noninvasive imaging, studies using anterograde and retrograde neuroanatomical tracers remain the key to obtaining high‐resolution maps of brain connectivity. Comparisons of results acquired using various diffusion tensor imaging (DTI) techniques against tracers have shown that current noninvasive techniques can only visualize the trajectory of axons that form relatively dense, ordered tracts in the white matter (e.g., Dauguet et al., 2007b; Thomas et al., 2014; Calabrese et al., 2015), a factor that excludes many of the connections between cortical areas. Moreover, the DTI techniques cannot yield information about the direction of the connections. Given that these limitations derive from basic biophysical factors, unlikely to be obviated in the near future, we have to rely on studies in animal models to obtain precise views of brain connectivity.

However, it is also important to recognize that much can be done to improve the way the information obtained in neuroanatomical tracing studies is stored and shared, including adoption of neuroinformatic procedures such as those developed for the field of human neuroimaging. Even today, a typical journal article reporting on the connections of a given area or nucleus illustrates a very small fraction of the collected material, leaving the vast majority of the data unavailable to other researchers. Perhaps just as important, selection of those materials that become "immortalized" in print is reliant on interpretations provided by the authors, which are necessarily based on the information available to them at a given point in time. Given the selective nature of what appears in print, and the lack of access to primary sources, it is rarely possible to use data collected by earlier investigators to test new hypotheses, refine models, or reconcile data from different laboratories. Indeed, in many cases the only way to advance knowledge is to obtain new datasets, a situation that is undesirable in terms of time, resources, and use of valuable animals.

In response to this challenge, there has been a trend towards the creation of open‐access repositories of data obtained by neuroanatomical tracing studies. Some of these rely on resources gathered via literature mining (e.g., Stephan et al., 2001; Bota et al., 2005), while others are based on processing primary data through high‐throughput microscopic imaging methods (e.g., Ragan et al., 2012; Mitra et al., 2013; Osten et al., 2013). The latter, in particular, have so far been introduced mainly to preserve and consolidate data about the mouse brain; these include the Allen Mouse Brain Connectivity Atlas project (Oh et al., 2014; http://connectivity.brain-map.org/), the Mouse Brain Architecture Project (http://brainarchitecture.org/), and the Mouse Connectome Project (http://www.mouseconnectome.org/). However, there are important differences between rodent and primate brains, particularly at the level of specific anatomical circuits such as those involved in the comprehension of complex visual and auditory patterns (e.g., faces and voices), and in the control of hand and eye movements (e.g., Rosa and Tweedale, 2005; Kaas, 2008; Wang et al., 2008; Kaas et al., 2011; Solomon and Rosa, 2014; Izpisua Belmonte et al., 2015; Mitchell and Leopold, 2015). To achieve an understanding of the anatomical bases of these and other functions, studies involving nonhuman primates are necessary. Yet, considering the brain volume of the most commonly used nonhuman primates (macaque monkeys), it is unlikely (on methodological, ethical, and economic grounds) that the same mass‐processing strategies developed for mouse brains can be simply scaled to produce a high‐resolution connectome.

One solution to alleviate this problem would be the creation of a computational procedure that is flexible enough to generate digital representations of full datasets of primate brain connectivity, using materials already available in laboratories around the world, as well as new materials obtained through standard neuroanatomical tracing techniques. The present article demonstrates that this is feasible, using methods largely derived from those developed for the field of human neuroimaging, which allow registration and visualization of datasets from multiple tracer injections in the cortex of marmoset monkeys. Considering that the marmoset brain has a mass that corresponds to 8% of the macaque brain, and 0.6% of the human brain (Stephan et al., 1981), focusing on marmoset brains has the added advantage of reducing the number of cases needed to achieve a detailed connectome, while still allowing study of many of the key anatomical features that make primate brains distinctive.

Marmosets are small monkeys (300–400 g adult weight), which show accelerated development in comparison with most other primates (e.g., Burman et al., 2007). Marmosets have well‐developed frontal and temporal lobes (e.g., Roberts et al., 2007; Burman and Rosa, 2009; Hung et al., 2015), a sophisticated visual cortex (e.g., Solomon and Rosa, 2014; Mitchell and Leopold, 2015), multiple cortical areas involved in planning and execution of movements (e.g., Bakola et al., 2015), and systems involved in deciphering complex patterns of vocal communication (e.g., Wang, 2013). However, the topology of the marmoset brain (in particular, the cerebral cortex) is much simpler than that observed in other commonly used primates, with a surface area of cerebral cortex that is less than one‐tenth of that in macaques (Chaplin et al., 2013a). Finally, marmosets are the first primate species for which stable transgenic lines have been established (Sasaki et al., 2009). The development of this technology, combined with the short reproductive cycle in this species, has led to the marmoset being described as a "biomedical supermodel" (Cyranoski, 2009), prompting projects for mapping its genome (Marmoset Genome Sequencing and Analysis Consortium, 2014), patterns of gene expression (e.g., Mashiko et al., 2012), and knockouts of genes involved in brain development (e.g., Okano and Mitra, 2014). Knowledge of the normal (wildtype) brain anatomy in the marmoset will be an important enabling resource to allow the interpretation of the results of projects involving transgenic modification, and many other future studies.

## MATERIALS AND METHODS

### Dataset and experimental procedures

The initial dataset used in the development of our procedure resulted from experiments conducted in nine marmoset monkeys. All experiments conformed to the Australian Code of Practice for the Care and Use of Animals for Scientific Purposes, and were approved by the Monash University Animal Experimentation Ethics Committee. Each of these animals received multiple (up to four) injections of fluorescent tracers, which were aimed at the approximate stereotaxic coordinates of subdivisions of the prefrontal cortex (Burman and Rosa, 2009; Paxinos et al., 2012). Based on histological examination, most of the injections used in the present analyses were located in the frontopolar cortex (area 10) and subdivisions of the caudal prefrontal cortex (areas 8aD, 8aV, and 8b according to the nomenclature proposed by Paxinos et al., 2012). Injections in adjacent areas in the same animals (areas 9, 46V, and 6DR) were also included (see, e.g., Burman et al., 2011, 2014). We chose these cases for the present analyses based on the quality of the injections and cell labeling (e.g., no involvement of white matter, and robust long‐distance label including the thalamus), and because they had already been independently analyzed in detail by traditional neuroanatomical methods, thus providing a valid basis for comparison between the results of the expert‐based and automated procedures (Burman et al., 2011; Reser et al., 2013). Nissl‐stained images of sections from each case, together with the positions of injection sites and retrogradely labeled neurons, are publicly available through the Marmoset Brain Architecture website (http://marmoset.braincircuits.org).

The surgical procedures have been described in detail previously (Burman et al., 2011; Reser et al., 2013). Intramuscular (i.m.) injections of atropine (0.2 mg/kg) and diazepam (2 mg/kg) were administered as premedication, before each animal was anesthetized with alfaxalone (10 mg/kg, i.m.) 30 minutes later. Dexamethasone (0.3 mg/kg, i.m.) and amoxicillin (50 mg/kg, i.m.) were also administered prior to positioning the animals in a stereotaxic frame. Body temperature, heart rate, and blood oxygenation (PO_2_) were continually monitored during surgery, and, when necessary, supplemental doses of anesthetic were administered to maintain areflexia. To place injections in different areas, small incisions of the dura mater were made immediately over the intended injection sites to limit exposure of the brain's surface. Tracer injections were placed in the same hemisphere in each animal.

Four types of fluorescent tracers were used: fluororuby (FR; dextran‐conjugated tetramethylrhodamine, molecular weight 10,000, 15%), fluoroemerald (FE; dextran‐conjugated fluorescein, molecular weight 10,000, 15%), fast blue (FB), and diamidino yellow (DY). The dextran tracers resulted in bidirectional transport, but only retrograde labeling is reported here. In most cases the tracers were injected using 25‐μl constant rate microsyringes (Hamilton, Reno, NV) fitted with a fine glass micropipette tip (see Table 1 for details). Each tracer was injected over 15–20 minutes, with small deposits of tracer made at different depths. Following the last deposit (typically at a depth of 300 μm), the pipette was left in place for 3–5 minutes to minimize tracer reflux. In some cases, FB and DY were directly inserted into the cortex as crystals (∼200 μm in diameter) with the aid of blunt tungsten wire (Rosa et al., 2005). Fluorescence microscopy photomicrographs of the types of injection sites and quality of the cell filling obtained with these methods are shown in recent publications from our laboratory (e.g., Palmer and Rosa, 2006; Burman et al., 2011, 2015; Reser et al., 2013). Estimates of injection extent for each case, drawn under microscopic examination, are available through the project's website.

**Table 1:** Details of the Injection Sites Analyzed

					Coordinates according to the automated procedure (mm)			Coordinates according to assessment by expert anatomist (mm)^a^			
	Case	Tracer	Full name of the structure	Abbreviation	M‐L	A‐P	D‐V	M‐L	A‐P	D‐V	Discrepancy (mm)
1	CJ70	DY	area 10 of cortex	A10	1.0	−19.5	11.9	1.0	−19.5	11.9	0.01
2	CJ70	FR	area 8a of cortex dorsal part	A8aD	3.9	−16.3	13.6	4.0	−16.0	13.0	0.66
3	CJ71	FE	area 10 of cortex	A10	0.6	−18.5	11.8	0.5	−18.5	12.1	0.32
4	CJ71	DY	area 10 of cortex	A10	1.4	−19.5	11.0	1.2	−19.5	11.1	0.22
5	CJ71	FB	area 10 of cortex	A10	2.8	−19.0	11.0	3.0	−19.0	11.5	0.52
6	CJ73	DY	area 9 of cortex	A9	0.6	−16.3	14.4	1.0	−16.5	13.6	0.96
7	CJ73	FB	area 46 of cortex ventral part	A46V	5.2	−16.8	13.2	5.1	−17.0	12.5	0.78
8	CJ73	FR	area 10 of cortex	A10	1.1	−18.4	10.7	0.7	−18.5	11.1	0.58
9	CJ74	DY	area 8b of cortex	A8b	0.2	−13.3	15.0	0.5	−14.0	15.0	0.79
10	CJ74	FB	area 8b of cortex	A8b	1.4	−15.2	14.2	1.5	−15.0	14.1	0.32
11	CJ75	DY	area 8a of cortex ventral part	A8aV	7.3	−15.0	12.4	7.0	−15.0	12.5	0.29
12	CJ83	DY	area 8b of cortex	A8b	0.9	−14.3	14.4	1.4	−15.5	14.3	1.31
13	CJ94	DY	area 8a of cortex ventral part	A8aV	6.6	−14.0	12.9	7.4	−14.0	11.8	1.36
14	CJ108	FE	area 8a of cortex ventral part	A8aV	5.8	−16.0	12.7	6.4	−16.0	12.5	0.66
15	CJ108	FR	area 8a of cortex dorsal part	A8aD	3.9	−15.4	13.7	4.0	−15.0	14.0	0.56
16	CJ125	FE	area 6 of cortex dorsorostral part	A6DR	4.1	−13.4	14.6	4.0	−13.5	15.0	0.47
17	CJ125	FR	area 8a of cortex ventral part	A8aV	6.7	−14.9	12.4	6.5	−14.5	12.6	0.44

a This column lists coordinates estimated by an experienced neuroanatomist (M.G.P.R.) based on comparison of sections from the animal with the plates provided by Paxinos et al. (2012). This assessment was made blind to the results of the automated procedure we report here.

The brains were separated into rostral and caudal blocks (to facilitate sectioning in a coronal plane), and five series of sections were obtained at 40 μm thickness. One in five sections was plotted under fluorescence microscope. Nissl staining was performed in sections adjacent to those used for fluorescence microscopy, as detailed in Burman et al. (2011) and Reser et al. (2013). Although other stains (myelin, cytochrome oxidase, and, in some animals, calbindin) were not relevant for the development of the automated workflow we report here; they were used in the manual (expert‐based) parcellation of the cortex that provided our basis for assessing the precision of the present method.

### Imaging and plotting

**Figure 1:**
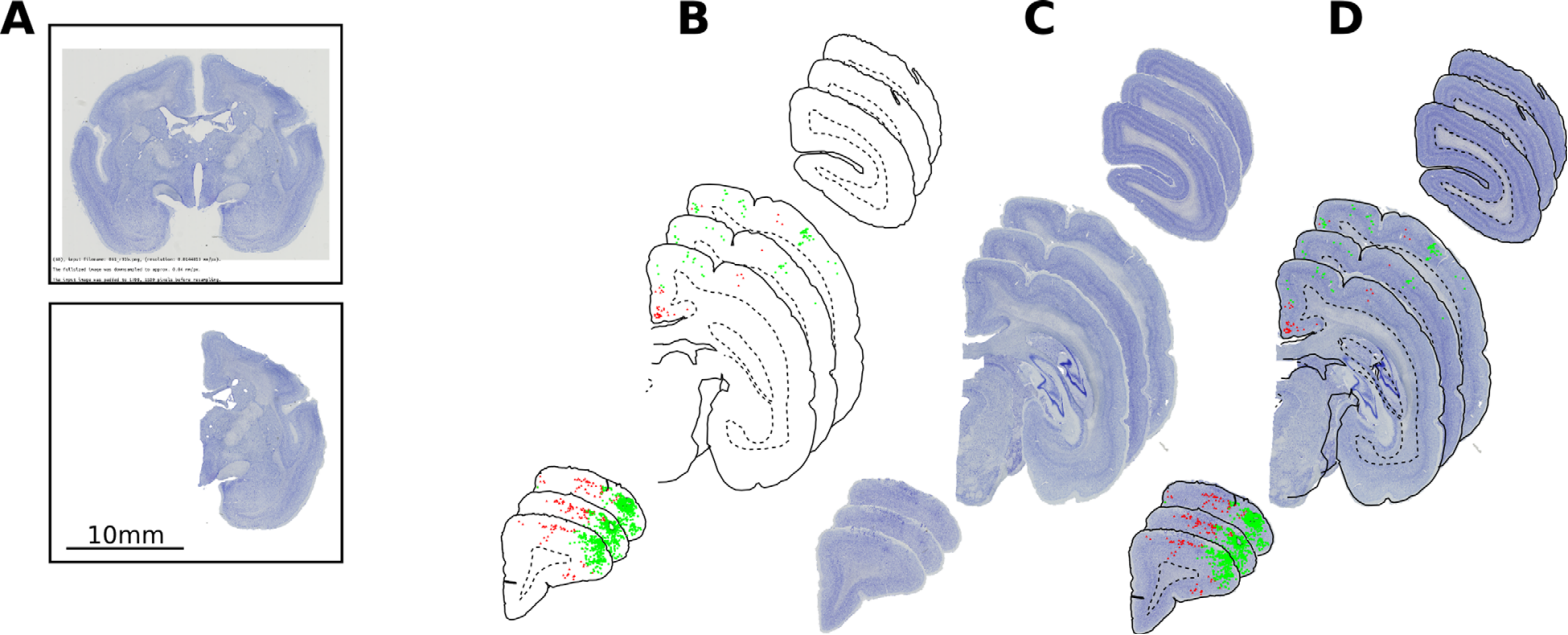
Preprocessing of the Nissl sections and plotting software data. **A**: Masking a left hemisphere on an example section. **B–D**: Alignment of the plotting software drawings based on fluorescent sections with corresponding Nissl‐stained sections. B: Plotting software drawings; individual colors denote different types of tracer, points correspond to the individual cells. C: Nissl‐stained sections corresponding to the fluorescent sections. D: Plotting software drawings aligned to the Nissl‐stained sections. [Color figure can be viewed in the online issue, which is available at wileyonlinelibrary.com.]

The Nissl‐stained sections from each case were scanned with a high‐resolution slide scanner (Nanozoomer 2.0 C9600‐12, Hamamatsu, Japan) at a resolution of 0.45 μm per pixel as 24‐bit RGB images. Since each slide contains multiple sections, each section was manually extracted and saved to a separate file (Fig. 1A). The Nissl method was chosen as the basis for the present procedure, as this is a commonly used method across laboratories, which provides consistent staining quality and results in high‐contrast images; however, the same procedure can, in principle, be adapted to registration of histological materials stained with other methods.

The sections used for analysis of tracers were examined using Zeiss Axioplan 2 or Axioskop 40 epifluorescence microscopes. First, the outline of the section and various brain structures were plotted using a digitizing system (MD3 digitizer and MDPlot software, AccuStage, Shoreview, MN) attached to the microscope, creating a line drawing of the section. Then labeled neurons were identified using ×10 or ×20 dry objectives, and their locations within the brain were plotted in the MDPlot drawing (Fig. 1B). To minimize the problem of overestimating the number of neurons due to inclusion of cytoplasmic fragments, labeled cells were accepted as valid only if a nucleus could be discerned. This was straightforward in the case of DY, as this tracer only labels the neuron's nucleus (Keizer et al., 1983). In the case of tracers that label the cytoplasm (FB, FE, and FR), the nucleus was discerned as a profile in the center of a brightly lit, well‐defined cell body, which in the vast majority of cases had an unmistakable pyramidal morphology. Because of the very dense labeling in the neighborhood of the injection sites, which resulted in difficulty in distinguishing cells labeled via axonal transport from those that incorporated the tracer passively, intrinsic connections were not included in the present quantitative analyses.

The extent of the DY or FB injection site was estimated according to the criteria defined by Condé (1987). For FR and FE injections, the injection sites were estimated as encompassing the neighborhood of the needle track and the immediate surround, where virtually every cell body was brightly labeled (Schmued et al., 1990). The volumes of injection sites are presented in Table 2.

**Table 2:** Volumes of the Injections Sites Analyzed

	Case	Tracer	Injection site volume (mm^3^)
1	CJ70	DY	0.2
2	CJ70	FR	0.4
3	CJ71	FE	0.2
4	CJ71	DY	0.2
5	CJ71	FB	0.4
6	CJ73	DY	0.5
7	CJ73	FB	0.7
8	CJ73	FR	1.1
9	CJ74	DY	0.1
10	CJ74	FB	0.5
11	CJ75	DY	0.4
12	CJ83	DY	0.7
13	CJ94	DY	0.3
14	CJ108	FE	0.2
15	CJ108	FR	0.2
16	CJ125	FE	0.2
17	CJ125	FR	0.6

### Preprocessing for automated workflow

The fact that the present workflow was designed with the use of archival materials in mind (particularly those collected without the intent of computerized processing) required the incorporation of a series of preprocessing steps, before the sections could constitute input to the reconstruction steps. The complete series of Nissl‐stained sections of each case was arranged in rostrocaudal order. The number of sections varied from case to case, to a maximum of 176 (case CJ70). The nominal section thickness was 40 μm; however, since the brains were prepared as five series, the apparent thickness amounted to 200 μm. The section images were downsampled to a resolution of 40 μm per pixel. Individual images were then inspected for quality and manually corrected as required. Typical corrections included: mirroring the image horizontally when the section was flipped during mounting, rotating to the upright position in case it was rotated more than 45°, and digitally rejoining displaced or detached parts; the latter was most commonly needed near the limit between the rostral and caudal blocks, where incomplete sections were in many cases obtained. Sections distorted beyond the possibility of correction (fewer than 5% of all sections) were replaced with their closest undistorted neighbors.

The images then underwent a masking procedure during which parts of the image representing brain tissue of a single hemisphere were selected, while the remaining voxels (contralateral hemisphere and the entire cerebellum) were discarded (Fig. 1A). The masking procedure was conducted using the open source ITK‐SNAP 2.4 application (Yushkevich et al., 2006; http://itksnap.org).

Since the locations of the tracer labeled cells are plotted on the fluorescence series, not the adjacent Nissl series, the MDPlot drawings must be aligned to their nearest Nissl section. The MDPlot drawing files were exported as a text‐based format (MDO) from MDPlot and parsed with custom Python programming language scripts to extract the section outlines and labeled cell locations (Fig. 1B). Then scalable vector graphics (SVG) files were generated, in which the MDPlot drawings were superimposed (Fig. 1D) on the Nissl section images (Fig. 1C). Each drawing was aligned to the Nissl section, which generally involved a simple set of translation, rotation, and scaling operations. Occasionally, manual editing of the drawing was required to make it correspond correctly with the Nissl section, typically in regions of the tissue that contained section folds and tears acquired during histological processing (e.g., if the section used for fluorescence plotting had partially detached from the slide).

### Marmoset brain template

**Figure 2:**
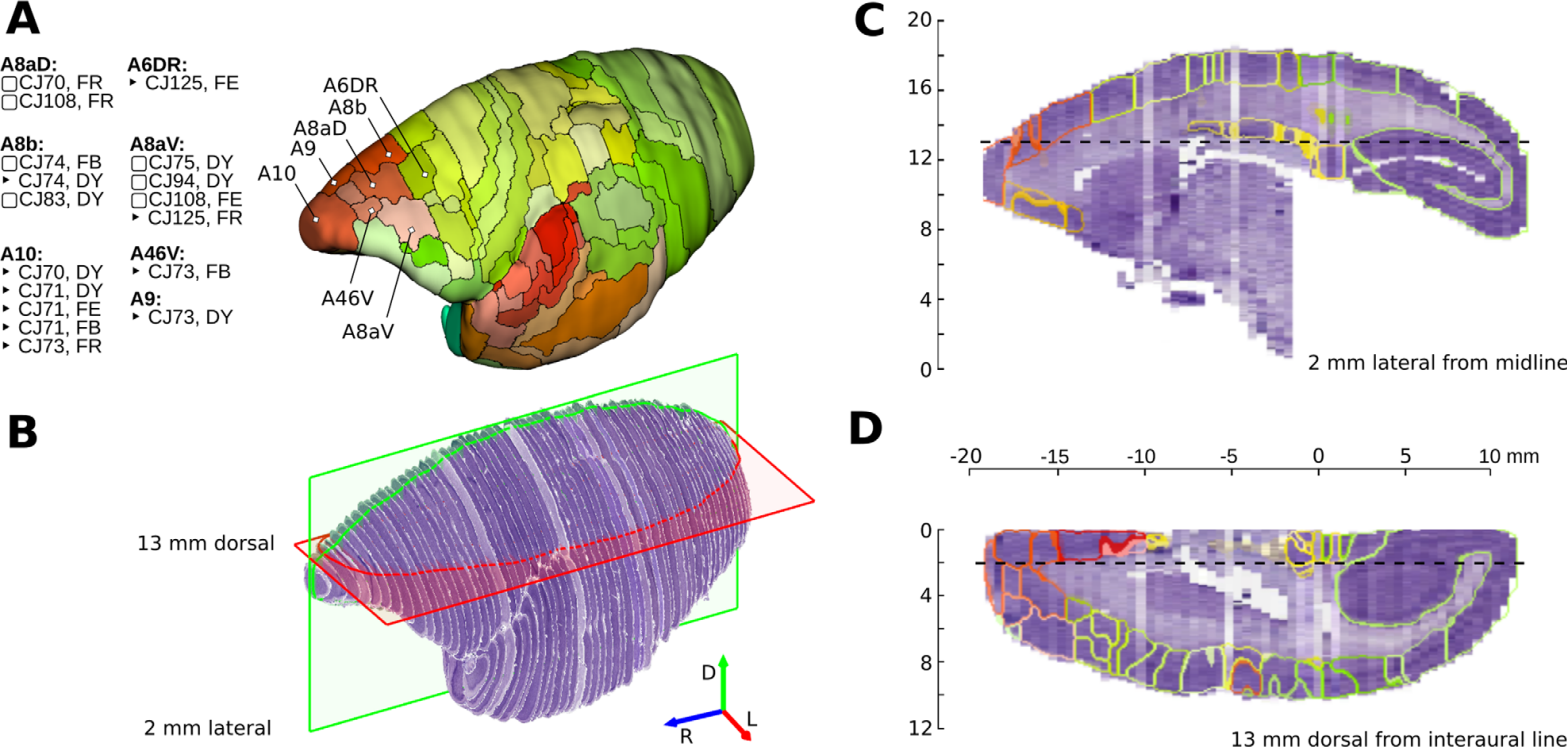
Reference template used in this study. **A**: Triangular mesh representing outline of the 3D reconstruction of the left hemisphere of the Paxinos et al. (2012) marmoset brain atlas. Individual colors correspond to different cortical areas. Cases and structures into which injections analyzed in this study were made are listed next to the mesh. A8aD: area 8a of cortex dorsal part; A6DR: area 6 of cortex dorsorostral part; A8b: area 8b of cortex; A9: area 9 of cortex; A10: area 10 of cortex; A46V: area 46 of cortex ventral part; A8aV: area 8a of cortex ventral part. Injections denoted with the square symbol were used for quantitative examination of the registration accuracy (Fig. 6B). **B**: 3D reconstruction of the brain template based on 63 Nissl atlas plates of the reference atlas. Green and red outlines correspond to sagittal **(C)** and horizontal **(D)** cross‐sections, respectively. C,D: Cross‐sections of the reconstruction shown in (B), colored contours represent atlas parcellation from (A). Axes indicate that the reconstruction is anchored in the stereotaxic reference system as defined in the Paxinos et al. (2012) atlas. The template is available as a Supplementary Material (Supplementary File S4_marmoset_brain_template.zip). [Color figure can be viewed in the online issue, which is available at wileyonlinelibrary.com.]

The objective of registering data from many different animals to a common 3D representation requires a template brain. This reference template was generated based on the electronic (PDF file) version of the Paxinos et al. (2012) marmoset brain atlas (available at: ftp://ftp.space.intersect.org.au/neura/), which contains a complete set of cortical areas based on histological examination. Delineations of 139 cortical areas were converted into a 3D image (Fig. 2A) using open‐source 3D Brain Atlas Reconstructor software (Majka et al., 2012; http://www.3dbar.org/) as described previously (Chaplin et al., 2013b). The nomenclature and abbreviations followed that of the atlas. Additionally, high‐resolution images of the Nissl‐stained sections of the atlas specimen were downloaded from an online repository (http://marmoset-brain.org/indexjp.html), aligned with the atlas parcellation, and reconstructed into volumetric form (Fig. 2B).

The resulting reconstruction was a 3D image with a resolution of 40 × 500 × 40 μm (mediolateral, rostrocaudal, and dorsoventral, respectively). The size of the image was 825 × 63 × 550 voxels (Fig. 2C,D). The reconstructed morphology is accompanied by the 3D‐labeled image of the brain parcellation of the same size and resolution. The 3D reconstruction preserves the exact stereotaxic coordinate system as defined in the atlas (Fig. 2C,D). This template is available in the Supplementary Materials attached to the present paper (Supplementary File S4_marmoset_brain_template.zip), and through the project's website (http://marmoset.braincircuits.org/static/S4_marmoset_brain_template.zip).

The cortical thickness of the template was measured using the Jones et al. (2000) algorithm. Additionally, normalized thickness was calculated so that 0 corresponds to the pial surface and 1 to the border between the white matter and the gray matter. A surface defined by points of normalized cortical thickness equal to 0.5 was extracted (the mid‐thickness surface) and converted to a flat map using the free, open‐source, software package CARET (Computerized Anatomical Reconstruction and Editing Toolkit, Van Essen et al., 2001; http://brainvis.wustl.edu/wiki/index.php/Caret: About) and the approach described previously (Reser et al., 2013; Burman et al., 2015).

### Computational environment

The 3D reconstruction process involved multiple steps and utilized several freely available open‐source software packages. The Possum reconstruction framework (Majka and Wójcik, 2015; https://github.com/pmajka/poSSum) provided computational pipelines for individual subtasks in the reconstruction of series of 2D images into 3D form. Image registration was performed by the Advanced Normalization Tools (ANTS) software suite (Klein et al., 2009; Avants et al., 2011; http://picsl.upenn.edu/software/ants/). ANTS allows one to conduct rigid and affine image registration as well as deformable image warping using the Symmetric Normalization algorithm (SyN, Avants et al., 2008) in both 2D and 3D images. Additional tools were used for creating, editing, and composing bitmap images: Convert3D (Yushkevich et al., 2006) and ImageMagick software (http://www.imagemagick.org/). 3D visualizations were prepared using a purpose‐written set of Python applications utilizing the Visualization Toolkit (Schroeder, 2006; http://www.vtk.org/) and the Insight Toolkit (Schroeder, 2005; http://www.itk.org/) biomedical image processing and visualization frameworks.

Based on this set of tools, a workflow was developed that allows one to conduct the reconstruction process in a streamlined and reproducible way. The computations were performed on the Massive 2 cluster (https://www.massive.org.au/) under GNU/Linux operating system deployed on a dual Intel Xeon E5‐2643 (16 × 3.30 GHz logical processors) nodes fitted with 32 GB of memory. The computational cost of the reconstruction was 200–240 CPU hours per case, depending on the number of sections.

### 3D reconstruction

**Figure 3:**
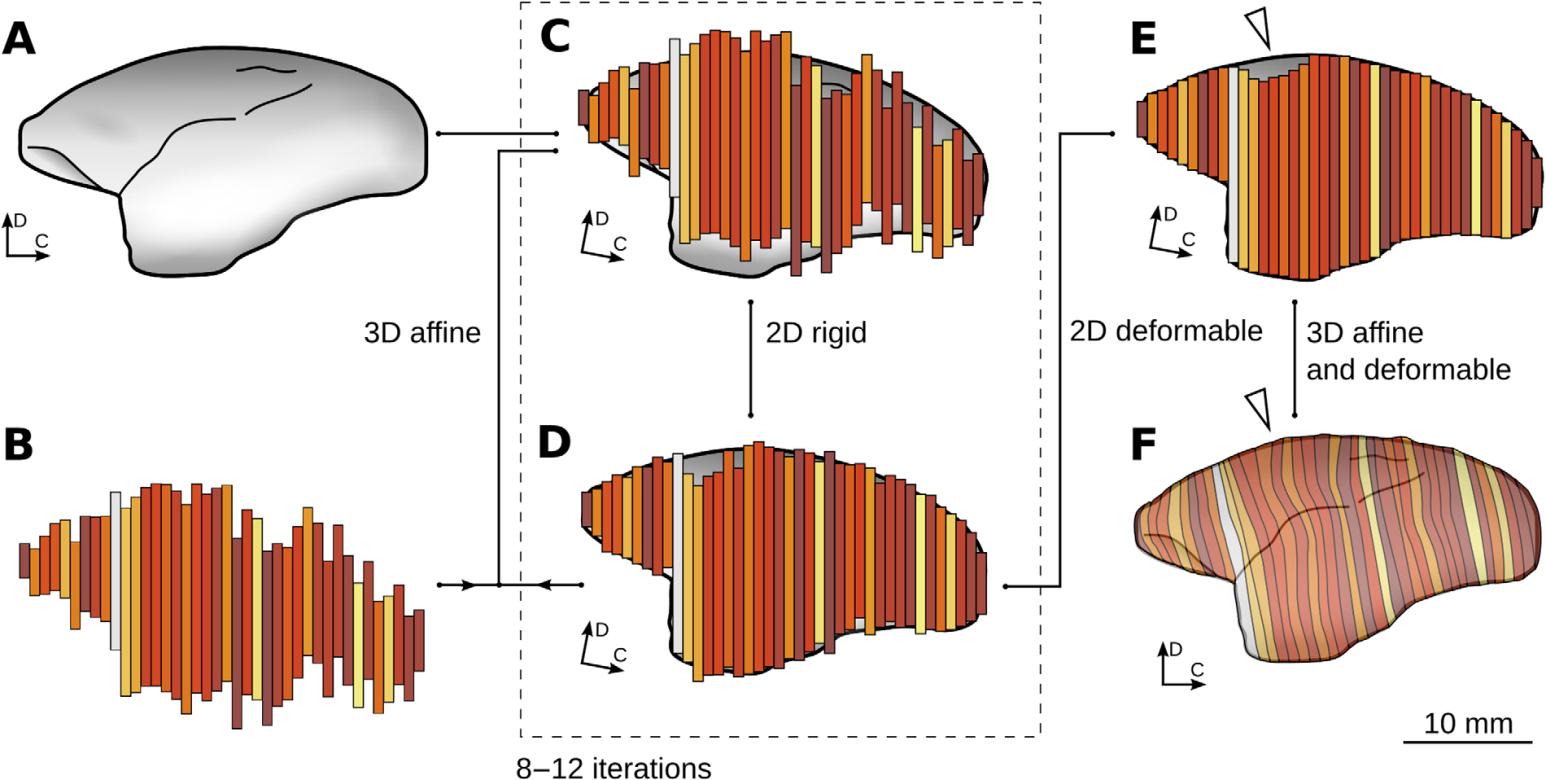
Reconstruction and normalization process. Note that in this illustration, for clarity, the process is represented in two dimensions. **A**: 3D image of the reference brain in Nissl modality (as shown in Fig. 2B). **B**: Series of the images of Nissl‐stained sections to be reconstructed and normalized (as shown in Fig. 1C), colored rectangles represent individual coronal sections viewed from a lateral viewpoint. **C**: The reference image aligned to the experimental stack using 3D affine transformation and resliced, so that each experimental section has its virtual reference cut assigned. **D**: Individual experimental sections are aligned to appropriate virtual references using 2D rigid (translation and rotation) transformation. Steps C and D are performed iteratively (dashed frame) and in each iteration agreement between reconstruction and the reference improves. **E**: A deformable reconstruction step enhances smoothness of the 3D image by accounting for uncorrelated distortions of individual sections. **F**: 3D affine followed by 3D deformable registration is used to warp the reconstruction (E) into the reference 3D image (A). [Color figure can be viewed in the online issue, which is available at wileyonlinelibrary.com.]

In order to coregister individual cases with the 3D template image, the set of 2D images from each case (Fig. 3B) must first be reconstructed into a 3D image with realistic, anatomical shape. This is achieved by performing two types of alignments alternately (Malandain et al., 2004; Yang et al., 2012). Given the reference atlas image (Figs. 2B, 3A) and the input stack of images (Fig. 3B), the process employs two complementary procedures:

1) It aligns the reference image to the stack using 3D affine transformation (Fig. 3C), after which each section from the input stack has its own virtual reference section assigned. This step accounts for the fact that the sections might have not been cut in the exact coronal plane as defined in the atlas.
2) Subsequently, the experimental sections are aligned to the virtual reference sections using 2D rigid transformation.

These two steps are repeated until obtaining convergence to a reconstruction which has a faithful anatomical shape of the brain, and in which the neighboring sections are affinely aligned to one another (Fig. 3D). Eight to twelve iterations were conducted, depending on the case. The Mattes Mutual Information (MI, Mattes et al., 2001) was used as an image similarity metric.

The next step was to use the deformable reconstruction scheme (Adler et al., 2014; Majka and Wójcik, 2015) to account for uncorrelated, section‐specific distortions of the individual sections, i.e., small amounts of compression, stretching, and bending of the tissue. The deformable reconstruction method stems from an assumption that variability of the shape of the brain structures is larger (i.e., it extends further) than the section thickness itself, thus the neighboring images are similar to one another in a formal sense (Adler et al., 2014; Gaffling et al., 2015). Therefore, the first stage of this process relies on warping each given section image towards an average image of its immediate neighbors in either direction. Such warping is performed for all images in the stack, which constitutes a single iteration of the procedure. Typically, eight iterations were performed (Fig. 3E). The SyN algorithm parameters used in the ANTS software in this process are provided in the Supplementary Materials (Supplementary File S1_reconstruction_and_normalization_parameters.pdf).

### Coregistration with the reference template

**Figure 4:**
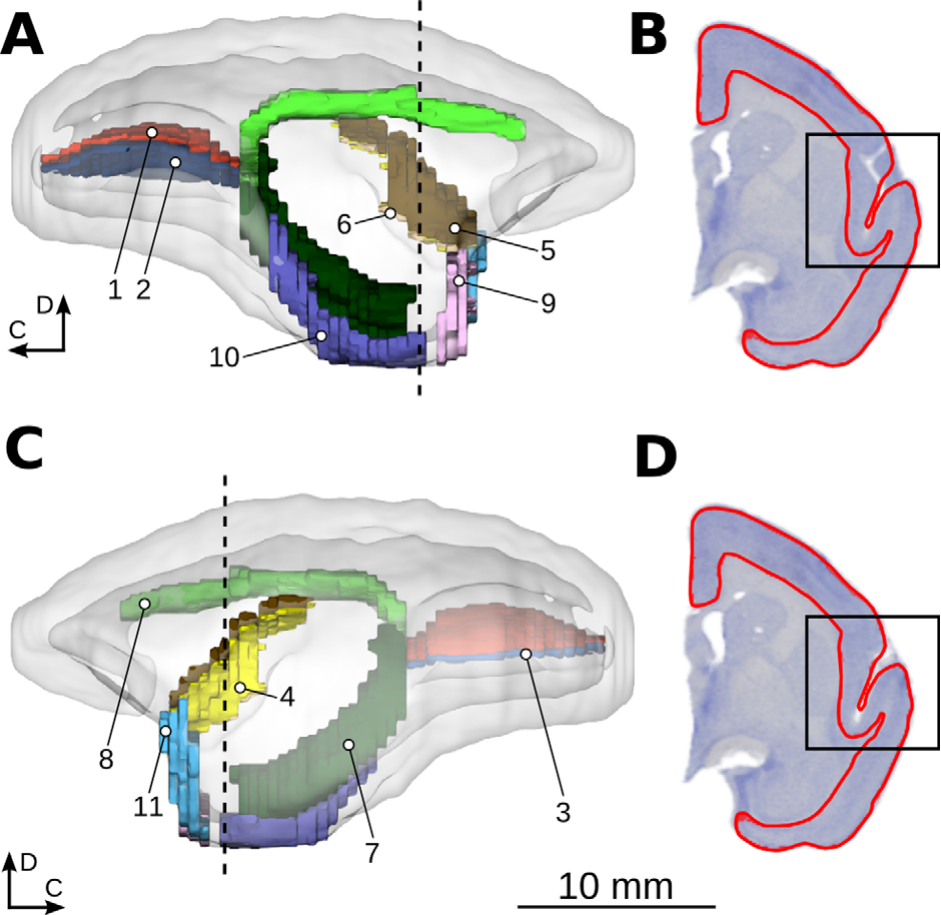
Individual sectors of cortical tissue were delineated to increase the coregistration accuracy. **A,C**: Medial and lateral views of the brain outline (gray, transparent model) with individual regions of cortex shown in different colors: dorsal bank (1), ventral bank (2) and fundus (3) of the calcarine sulcus, lateral bank (4), medial bank (5) and the fundus of the lateral sulcus (6), hippocampus (7), cingulate cortex (8), enthorinal cortex and parasubiculum (9), piriform cortex (10), and isocortex of the temporal pole (11). Dashed lines denote the coronal plane shown in B,D. **B,D**: Coronal sections through the 3D brain image of case CJ83, coregistered without incorporating the information on corresponding tissue fragments (B) and using such information (D); red outline denotes contour of the cerebral cortex as defined in the atlas, while the black rectangles highlight an area within which the difference between the two coregistration attempts is the most noticeable. [Color figure can be viewed in the online issue, which is available at wileyonlinelibrary.com.]

First, both the 3D reconstructions and the atlas image were preprocessed by resampling to an isotropic resolution of 75 μm and smoothing with a median filter with a 1 voxel radius. Next, ITK‐Snap was used to delineate 11 sectors of cortical tissue landmarks (Fig. 4) of the cerebral cortex in both the reconstructed 3D image (Fig. 3E) and the atlas image (Fig. 3A). The landmark structures were as follows: dorsal bank (1), ventral bank (2), and fundus (3) of the calcarine sulcus, lateral bank (4), medial bank (5), and the fundus of the lateral sulcus (6), hippocampus (7), cingulate cortex (8), enthorinal cortex and parasubiculum (9), piriform cortex (10), and isocortex of the temporal pole cortex (11).

The process of coregistration with the atlas starts with the affine transformation followed by deformable warping. The latter process was driven by two similarity metrics: cross‐correlation coefficient (CC, Avants et al., 2008) with a kernel size of 5 voxels, and the Point‐Set Expectation (PSE, Avants et al., 2011) metric, which forces corresponding landmarks to overlap. Both metrics were used with equal weights. Subsequently, the experimental 3D image is warped using the calculated transformations so it matches the atlas (Fig. 3F).

### Mapping the locations of individual labeled cells into the reference template

The process of reconstruction and normalization results in a 2D affine and a 2D deformable transformation for each section, as well as a 3D affine transformation and a 3D deformable displacement field for each case. This set of transformations provides a way for expressing location of every point in the raw histology stack in the coordinate system of the atlas. Using these transformation locations of cells and injection sites were mapped into the template.

Based on the atlas parcellation, each cell and injection site were assigned to a cortical area. Points which were mapped outside the delineation of the cortical areas were assigned to the nearest cortical structure. Each point was also assigned with a nominal and normalized depth below the pial surface, or values of 0 or 1, if it was located outside the brain or in the subjacent white matter, respectively. This process was applied to histological data from nine specimens, which included 17 individual injections comprising ∼84,000 labeled cells.

### Cell mapping accuracy assessment

Several methods were used to evaluate the accuracy of the mapping procedure. The first was to determine if single, well‐defined points (the centers of mass of the injection sites) mapped to the same cortical areas that were determined by expert neuroanatomists, based on inspection of the histology. The second method was to measure the Euclidean distance between the location of the center of the injection site obtained from the mapping procedure to that estimated by an expert neuroanatomist, based on comparison between sections from a case and the plates provided by Paxinos et al. (2012). The third method was to compare the percentage of cells per cortical area obtained by the automated mapping versus the expert‐based cell count established by neuroanatomists, as reported previously in tabular form (e.g., Burman et al., 2011; Reser et al., 2013). The numbers of cells per single cortical area were grouped to match the reference data; for example, in the present cases several small auditory areas were grouped as a supergroup "core and belt," due to low cell counts (e.g., Reser et al., 2013). The percentage of cells in these studies was determined excluding cells located in the area into which the injection was made (intrinsic connections); thus, the percentage of cells in these structures is undefined. The expert‐based percentage of cells in structures with low cell count (0.05 to 0.1%) has been reported as a single value in the original studies. Finally the Pearson's correlation coefficient was calculated between the cell percentages obtained from seven injections into area 8 complex (CJ70–FR, CJ108–FR, CJ74–FB, CJ83–DY, CJ75–DY, CJ94–DY, CJ108–FE) denoted with the square symbol (□) in Figure 2A. In the case of comparisons involving injections in area 10 (Burman et al., 2011), the nomenclature of cortical areas was updated to bring the dataset in harmony with the more current designations proposed by Paxinos et al. (2012).

## RESULTS

### Reconstruction and normalization workflow

**Figure 5:**
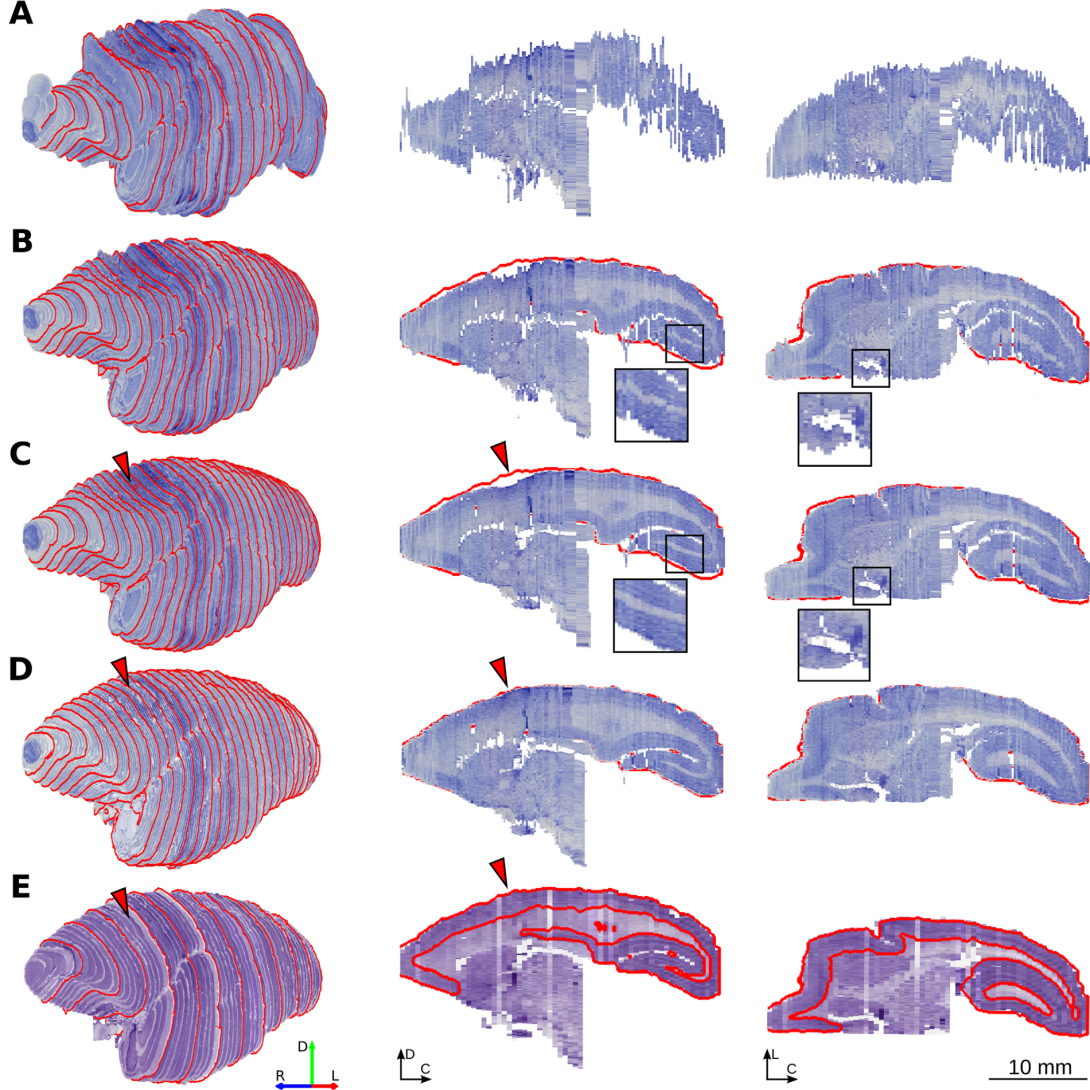
Consecutive steps of the 3D reconstruction and spatial normalization process of an example case (CJ94) comprising 159 images of Nissl‐stained coronal sections. **A**: Input images in which only the left hemisphere to be reconstructed was represented. **B**: Reconstruction after applying affine transformation. **C**: Reconstruction after applying deformable corrections. **D**: Final reconstruction matching the reference image. **E**: The reference template image. Columns: Left: Stacks of the Nissl‐stained sections viewed at 30° angle rostrolaterally. Every fifth section is outlined with a red contour to illustrate the general shape of the image stack. Middle: parasagittal cross‐sections. Right: Horizontal cross‐sections. See text for a detailed description of individual reconstruction steps. [Color figure can be viewed in the online issue, which is available at wileyonlinelibrary.com.]

The pipeline described above allows the processing of a stack of images of Nissl‐stained sections from any marmoset brain into a 3D form that matches that derived from the common marmoset brain atlas (Fig. 5). Three main steps can be distinguished: The affine reconstruction step (Fig. 5B), the deformable reconstruction step (Fig. 5C), and the combined, affine plus deformable, coregistration with the template image (Fig. 5D).

The stack of the input images (Fig. 5A) comprises individual sections arranged in rostrocaudal order. The right hemisphere in this case was masked out and only the left hemisphere was processed. This stack lacks spatial consistency since the sections are not aligned to one another.

The affine reconstruction step transforms the input stack so it takes the approximate anatomical shape of the reference brain, with consecutive sections affinely aligned to one another (Fig. 5B). This can be noticed by comparing sagittal and horizontal cross‐sections of the reconstruction with the outline of the atlas (Fig. 5B, middle and right columns). However, at this stage there are still noticeable sharp transitions between consecutive sections caused by tissue distortions, which naturally occur during the processing of the frozen sections (insets in Fig. 5B,C).

The subsequent deformable reconstruction step (Fig. 5C) mitigates the section‐specific distortions. The discontinuities in the transitions are largely eliminated, the overall appearance is much more natural (Fig. 5C, cross‐sections and insets), and details of brain anatomy are easier to perceive. This can be noticed by comparing, for instance, the course of the calcarine sulcus between the affine and deformable reconstructions (insets in Fig. 5B,C). This stage does not, however, eliminate global distortions, which occur as a consequence of the surgical process and further preparation of the brain tissue. For instance, a depression in the dorsal surface of the frontal lobe (red arrow in Fig. 5C) near the injection sites, caused by the surgical procedure, can still be noticed.

The last stage, the coregistration with the atlas, brings the reconstruction (Fig. 5C) into a precise match with the template image (Fig. 5E). This stage accounts for global distortions such as depressions or lesions. Additionally, the overall shape of the brain is slightly altered to match the reference image (red outlines in the horizontal and sagittal cross‐sections of Fig. 5C,D). The continuity of internal structures' shapes is also improved. Overall, this process establishes a mapping between points in the experimental dataset and the template, which allows one to transfer the spatial information from the Nissl sections of a specific animal to the atlas space.

### Use of landmarks

The use of landmarks improves the overall registration accuracy by significantly reducing the likelihood of coregistration algorithm falling into local minima of the image similarity function. This can be seen in Figure 4B, where the lateral bank of the lateral sulcus of the reconstruction is misaligned, and does not match its counterpart in the atlas. With the information provided by the landmarks a more accurate mapping is produced, as the process aligns actually corresponding image features (Fig. 4D). While in our materials the example of the lateral sulcus is the most evident situation in which the use of anatomical landmarks improves the registration (as the exact location and depth of this sulcus varies from animal to animal), there were several other regions of the cortex where this type of misregistration tended to occur. Although in the nine reconstructed cases reported in this article the landmarks were drawn manually, it is feasible to use image processing techniques, such as feature detection, to automate the process of drawing the landmarks in the future, thus making it user‐independent and less time‐consuming.

### Injection sites agreement

The automated procedure assigned the location of each injection site to a cortical area, based on registration to the delineations illustrated by Paxinos et al. (2012) for the brain of one extensively studied animal. An obvious important question in assessing the value of this method (and, more generally, any neuroinformatics procedure based on registration to a template brain) is how often is this automatic assignment correct. We assessed this by comparing the locations of the injection sites determined automatically with those established by an experienced neuroanatomist, who considered Nissl‐stained sections from the same animal as well as adjacent sections stained for myelin and cytochrome oxidase. This expert‐based assessment was conducted several years prior to the development of the present method (Burman et al., 2011; Reser et al., 2013). The comparison below quantifies the match between the manual and automated procedures for the 17 injections examined in this article.

**Figure 6:**
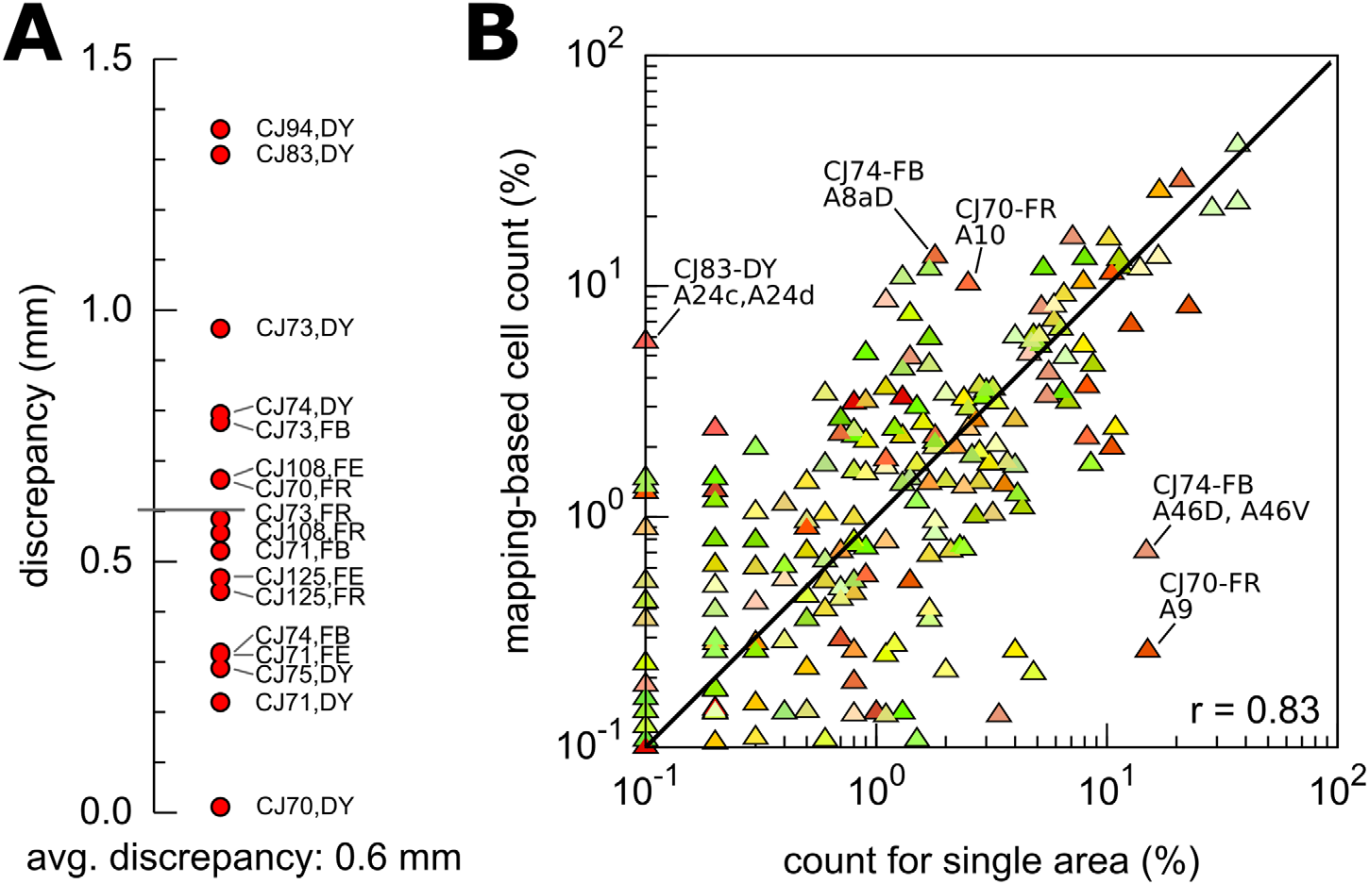
Cell mapping accuracy assessment. **A**: Euclidean distances between the locations of the injection sites obtained from the automated mapping procedure and those estimated by an expert neuroanatomist (by comparing individual sections from a case with the nearest matching plate from the Paxinos et al. (2012) reference atlas). **B**: Comparison of percentages of cells mapped into individual cortical areas by the automated (ordinates) and manual (abscissas) procedures. The black line denotes a line of an exact agreement between results obtained from both approaches. Colors of different points correspond to individual cortical areas as shown in Figure 1A. [Color figure can be viewed in the online issue, which is available at wileyonlinelibrary.com.]

The examination of the Euclidean distance between the locations of the estimated centers of mass of the injections, obtained by the two procedures, varied between cases (Fig. 6A), with values ranging between 0.01 mm and 1.36 mm. The average distance between the reference injection points and those obtained by the automated mapping amounted to 0.6 mm. The highest discrepancies were observed for cases CJ94–DY (1.36 mm, injection into area 8aV) and CJ83–DY (discrepancy of 1.31 mm, injection into area 8b). For a majority of the injections the discrepancy was in the range of 0.4–0.8 mm. Low discrepancies were observed for cases CJ71–DY (area 10 injection, discrepancy of 0.22 mm), CJ75–DY (injection into area 8aV, discrepancy of 0.29 mm), and CJ71–FE (0.32 mm, area 10). The CJ70–DY injection had the lowest discrepancy (0.01 mm); however, it has to be noted that this injection was made exactly in a frontal pole, which is a very distinctive landmark and thus coregistered well. No dependencies were identified between the amount of discrepancy and tracer type, or injected area. Importantly, in all of the cases processed the assignment of an injection to a specific cortical area coincided between the automated and expert‐based procedures.

### Comparison of the percentages of labeled cells in individual areas

A second important question that is common to any analysis of connectivity based on registration to a template brain is how often are the origins (or terminations) of neural pathways identified correctly. Given that for the present injections we had access to counts of labeled neurons in different areas (Burman et al., 2011; Reser et al., 2013), we were also able to assess the performance of the automated procedure by comparing the percentages of labeled neurons it assigned to different areas with these expert‐based data (Fig. 6B).

In a comparison of 546 pairs of expert‐based and automated procedure‐based values, we found that the Pearson's correlation coefficient was 0.83, revealing a good degree of correspondence. However, there were outlier points with high discrepancy (labeled points in Fig. 6B). For instance, in case CJ83–DY areas A24c and A24d had very few connections with area 8b in the reference data (0.1% of the extrinsic projections), while the automated procedure yielded an estimate of 5.7%. Another example is the percentage of labeled cells in area 9 in case CJ70–FR (area 8aD injection), in which the expert‐based estimate was 15%, while the automated mapping yielded 0.26%. Probable reasons behind such high discrepancies are discussed below (see *Limiting factors and proposed future developments*). The data used in the analysis shown in Figure 6B are provided in the Supplementary Materials (Supplementary File S2_cell_mapping_accuracy_assesement_results.pdf).

The number of connections for which the expert‐based estimate of the percentage of labeled neurons was equal or lower than 0.05% was 277. For 229 of these (83%) the mapping‐based percentage also yielded an estimate equal or lower than 0.05%. Similarly, the number of areas in which no labeled cells were found was 229 according to the expert‐based data, and 177 according to the automated approach (77%). Thus, while most sparse connections appear to be well characterized by the automated method, the use of a template in which areas have discrete (nonprobabilistic) boundaries results in assignment of some of the sparse connections to adjacent areas.

### Analysis and visualization capabilities

**Figure 7:**
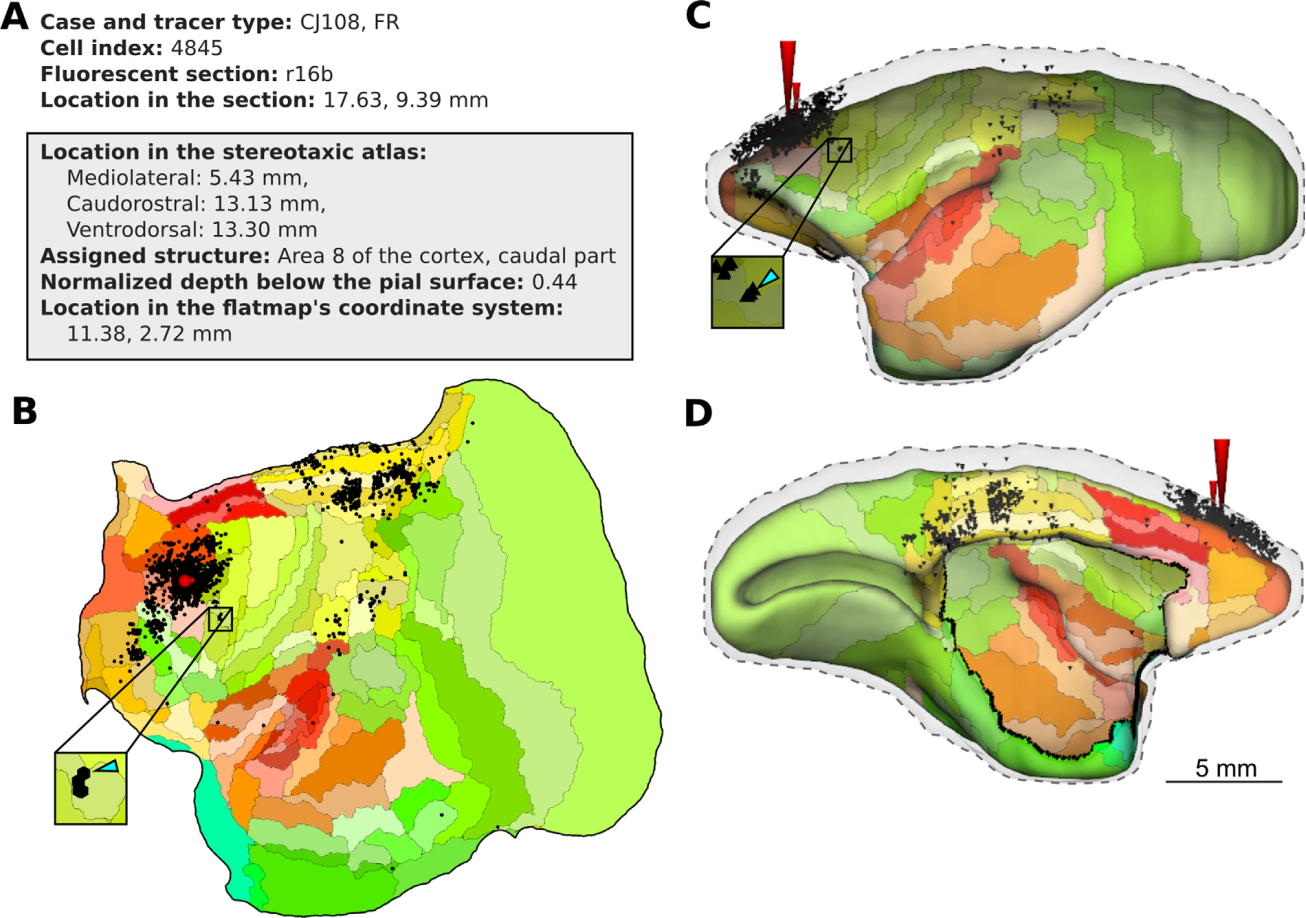
An example of 3D and flat map visualization of the cell mapping results (case CJ108FR, injection in the caudal part of area 8aD). **A**: An example database entry on a single cell showing data associated with the cell. The values within the gray rectangle are established during the mapping process. Flat map **(B)**, lateral **(C)**, and medial **(D)** views on the individual cells (black points) plotted against the mid‐thickness cortical surface (C,D) or the flat map (B). The tip of the large vertical red cone denotes the center of mass of the injection, and the small red cone the estimate obtained based on comparison with atlas plates by an expert neuroanatomist. Cell indicated with the blue wedge within the inset is the one detailed in A. In C,D, the dashed gray outline depicts the external surface of the cerebral cortex. [Color figure can be viewed in the online issue, which is available at wileyonlinelibrary.com.]

**Figure 8:**
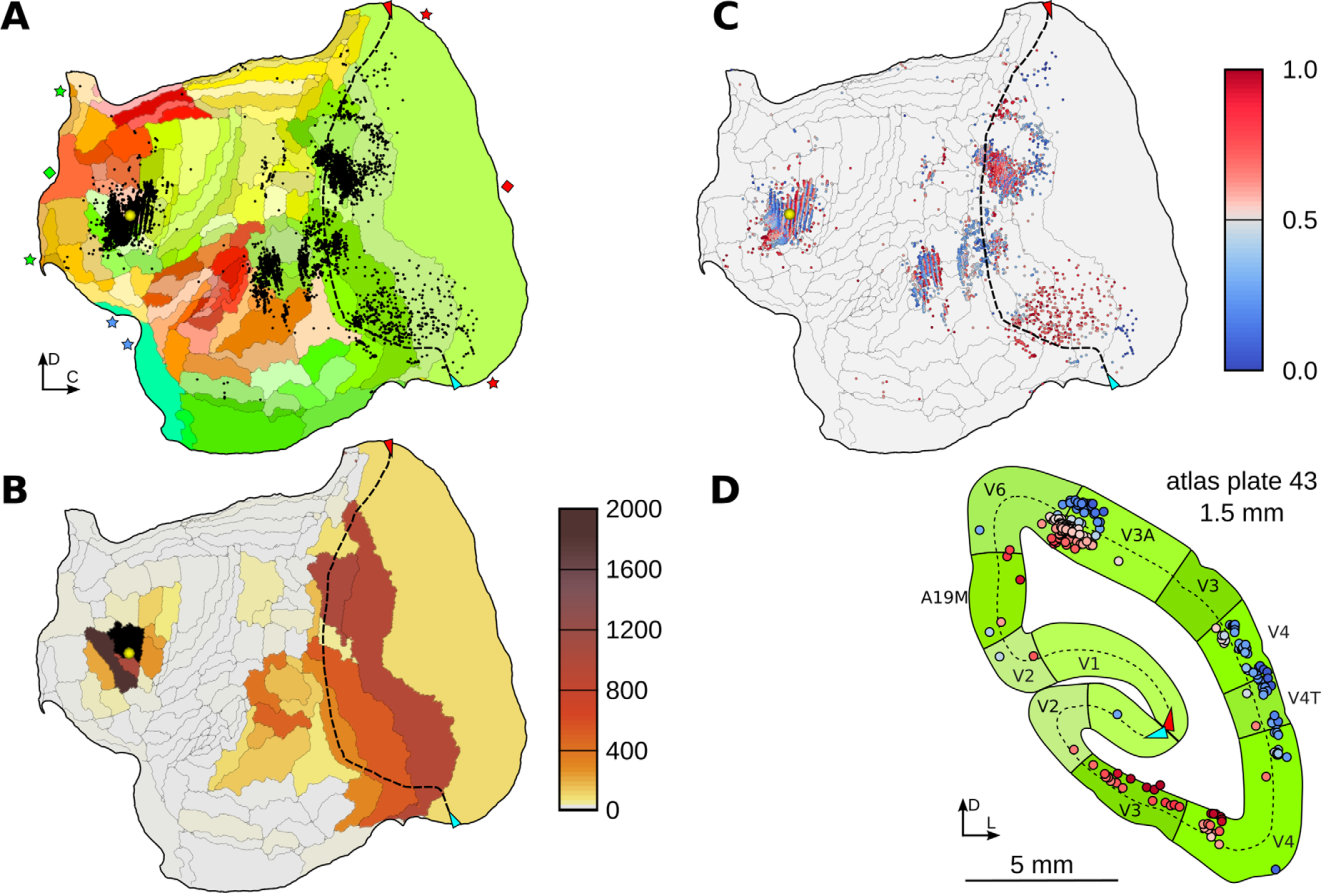
Visualizations of the pattern of cortical labeling resulting from the area 8aV injection in case CJ75DY, using the flat map representation of the atlas. **A**: Flattened mid‐thickness cortical surface. Different colors correspond to various areas, following the convention shown in Figure 6B. The green and the red diamonds denote the rostral and caudal poles, respectively, and the pairs of asterisks of same colors denote adjacent points, which became separated in the maps by introduced discontinuities, generated to reduce distortions resulting from the flattening procedure. Black dots denote individual cells projected onto the flat map, while the yellow sphere indicates the injection location. **B**: The numbers of cells assigned to different cortical areas (see the color scale). The area comprising the injection site (area 8aV, part of the frontal eye field; Burman et al., 2006) has an undefined number of cells. **C**: Normalized depth below the pial surface of individual cells. Cells placed close to the surface appear blue, while those lying close to the white matter boundary are red. **D**: Cells assigned to rostrocaudal coordinates between 1.3 mm to 1.7 mm caudal to the interaural line, plotted against plate 43 of the Paxinos et al. (2012) atlas. The colors indicate normalized depth below the cortical surface. The dashed black curve represents the mid‐thickness line. A corresponding dashed line is drawn in A–C, indicating the level of this section in the normalized flat map. The red and blue triangular markers show the direction of the mid‐thickness line, and indicate the fact that the calcarine sulcus was discontinued along its fundus. [Color figure can be viewed in the online issue, which is available at wileyonlinelibrary.com.]

The reconstruction and normalization process resulted in a database of cells and injection sites comprising the following parameters: identifier of the animal, type of tracer, atlas coordinates (lateral, rostrocaudal, and dorsoventral), cortical area, nominal and normalized distance below the pial surface, and 2D coordinates of the point after projection onto the flat map. An example entry from the database is shown in Figure 7A. The results may be utilized in several kinds of analyses (e.g., to calculate cell counts per cortical area, or to compute cell density) and to visualize the mapping of labeled neurons in the reference (atlas‐based) coordinate system. For instance, they can be plotted in 3D space against the reference template (Fig. 7C,D). Another way of visualizing the results is by generating flattened, mid‐thickness representations of the cortex, as commonly used to report results of the tracer injection studies (e.g., Rosa et al., 2009; Bakola et al., 2010, 2013; Passarelli et al., 2011; Burman et al., 2014, 2015). With this representation one can summarize, for example, the distribution of the cells across the cortex (Figs. 7B, 8A), their normalized depth relative to the pial surface and bottom of layer 6 (Fig. 8C), or the number of cells per cortical area (Fig. 8B). Due to the spatial normalization of the data one can also traverse between the representations (e.g., dashed line in Fig. 8C,D).

## DISCUSSION

### Reconstruction and normalization process

We have created a computational pipeline for 3D reconstruction and spatial normalization of data from retrograde tracer injections in cortex into a reference template of the marmoset brain. The process relies on multistage affine and deformable image registration steps, and ultimately results in a series of spatial transformations that allow one to express cell locations from different animals in a common set of stereotaxic coordinates (Fig. 2). The results thus produced can be quantified, analyzed, and visualized in various ways (Figs. 7, 8). The structure and complexity of the present workflow is comparable to similar procedures previously used, for instance, in human (e.g., Amunts et al., 2013; Annese et al., 2014), nonhuman primates (e.g., Choe et al., 2011; Dauguet et al., 2007a), and rodents (e.g., Ng et al., 2009; Yang et al., 2012). We see the reliance on well‐established and validated procedures and freely available open‐source software for volumetric reconstruction as one of the strengths of the current approach, as it may facilitate future integration with work in other species. However, the present study is novel in that it demonstrates the feasibility of integration of volumetric reconstruction from serial sections with data from retrograde tracer injections, in a primate, including normalization to a template. Registration of data from many individuals to a "template brain" is a common feature of most, if not all, current brain‐mapping projects. As part of the study, we also provide what we believe are the first estimates of the precision of this approach against traditional, expert‐based cell‐counting methods, in terms of errors incurred in quantifying specific connections between cortical areas.

Another point of distinction in the present approach was that it was developed with a focus on applicability to archival materials, in the form of sections collected and analyzed independently using traditional neuroanatomical techniques. Our main objective here was to allow future public releases of materials from a large number of such cases already collected in our laboratory, in addition to those already available in the Marmoset Brain Architecture website (http://marmoset.braincircuits.org). This may allow analyses by other researchers, using more sophisticated analysis techniques than those used in the original reports, and/or model‐based approaches (e.g., Chaudhuri et al., 2015). Although the present study was based on manually plotted fluorescent tracers, the proposed pipeline is versatile, and with only minor modifications can be adapted to use other sources of cell locations. For instance, software for automated detection of labeled cells, such as the Cell Profiler (Carpenter et al., 2006), Farsight Toolkit (Bjornsson et al., 2008), or Elastix (Sommer et al., 2011) might be utilized similarly, as in pipelines recently introduced for mice (e.g., Vousden et al., 2014). In addition, the volumetric reconstructions based on the proposed workflow may prove useful in allowing direct comparisons with other marmoset brain connectivity studies, using different imaging techniques (e.g., Okano et al., 2015). Attempts of such comparisons have been done, for example, for mice (Calabrese et al., 2015) and macaque (Dauguet et al., 2007b), which showed significant discrepancies between data obtained from tracer injections and DTI. This, in the future, can help to better interpret the connectivity patterns in human, where invasive connectivity studies cannot be performed. In addition, the conversion from serial sections to a volumetric representation that matches a reference brain can be applied to reconstructions based on images of sections obtained by sectioning the brain in planes other than coronal (e.g., horizontal, parasagittal, or oblique), and allows visualization of the data using different section planes (Fig. 5) or flat maps (Fig. 8). Finally, the computational solutions developed for the purpose of mapping locations of retrogradely labeled neurons in the cerebral cortex can, in principle, be incorporated in future developments such as mapping projections from subcortical structures, or the location of axonal pathways and terminal label resulting from anterograde tracer injections. Although the latter can be reconstructed and visualized across cases using the same workflow, it raises specific challenges in terms of quantification (Kuan et al., 2015).

The ultimate aim of developing our workflow is to create a platform for efficiently comparing and analyzing patterns of connections in a model primate brain. The present effort has to be seen in the context of parallel efforts to curate patterns of connections in the macaque brain, such as literature‐based CoCoMac database (Stephan et al., 2001; Bakker et al., 2012), and recent studies by Markov et al. (reviewed in Markov et al., 2013), which present data from a number of animals as Supplementary Materials. The CoCoMac database allows for coordinate‐free and parcellation‐based data representation, which is a complementary approach to the spatially based representation presented in the present article. It fosters cross‐species and cross‐atlases comparison, an aspect that will need to be incorporated in future developments focused on the marmoset. Even though the Markov et al. (2014) studies represent the best effort to date to amass data on the connectivity of the macaque cortex, at the moment the data cannot be publicly accessed in a format that allows flexibility in the analysis, and only selected sections are available as Supplementary Materials in the published articles. As an initial step towards full sharing of the data that form the basis of the present study, we have made the histological sections and locations of labeled neurons and injection sites available through a website (http://marmoset.braincircuits.org/).

Methods such as CLARITY (Chung et al., 2013) or other high‐throughput techniques, reviewed in Osten et al. (2013), can also be, in principle, applied in future studies of the marmoset brain. An approach similar to ours will nonetheless need to be integrated to allow for ready comparison between cases, and registration to histological subdivisions.

### Cell mapping accuracy

The reliability of the workflow was assessed initially by comparing stereotaxic coordinates of the injection sites estimated by the automated procedure, with those determined manually. The average discrepancy (0.6 mm) is close to the distance between consecutive atlas plates (0.5 mm), which indicates that at least some of the imprecision can be traced to limitations of the template itself (see detailed discussion below). It should be noted, however, that this discrepancy is not necessarily a reflection of imprecision in the registration procedure. Obvious factors that are likely to contribute to this figure include the subjective nature of the expert‐based estimates of the location of the injection site, by visual inspection and comparison with a stereotaxic atlas, and the simplification of the injection site into a discrete point, placed in the estimated center of mass of the injection. In our sample, the largest discrepancies observed between injection site coordinates according to the automated and expert‐based estimates corresponded to injections in the flat dorsolateral surface of the frontal lobe, away from prominent landmarks that would help a human observer to pinpoint coordinates with accuracy. Moreover, in at least one of these cases (CJ83; see Supplementary Fig. S3), the plane of sectioning was significantly tilted relative to the atlas plates, a factor that can affect human estimates, but is accounted for the by volumetric reconstruction procedure.

A second form of validation was the comparison of the numbers of cells in each cortical area, as estimated by the automated approach, with cell counts determined manually by experienced neuroanatomists, who visually inspected the cytoarchitecture of the relevant sections to obtain traditional delineations of areas. Inspection of Figure 6B suggests that the automated procedure replicates most of the main features of cortical connectivity that are apparent from the assessment by an expert (Pearson's correlation coefficient of 0.83), but also highlights discrepancies. An obvious reason for the latter is the fact that the exact sizes and shapes of cortical areas may vary between individuals, and thus labeled cells located in one area may be mapped into an adjacent area by the automated procedure. However, caution is required in interpreting the exact significance of these mismatches. Margins of error of 1 mm or more are common in the expert‐based approach, due to factors intrinsic to histological inspection (e.g., whether a border occurs within the same section, or between sections, whether a single section contains layers from adjacent areas, and possible staining artifacts). Importantly, with notable exceptions (e.g., primary sensory fields), the nature of the histological transitions between most subdivisions of the primate isocortex is best characterized as gradual, and delineations are often open to interpretations by different experts (for discussion, see Rosa and Tweedale, 2005). Thus, the "true" level of discrepancy introduced by a fully automated procedure may be less significant than that suggested by the type of comparison we used here.

**Figure 9:**
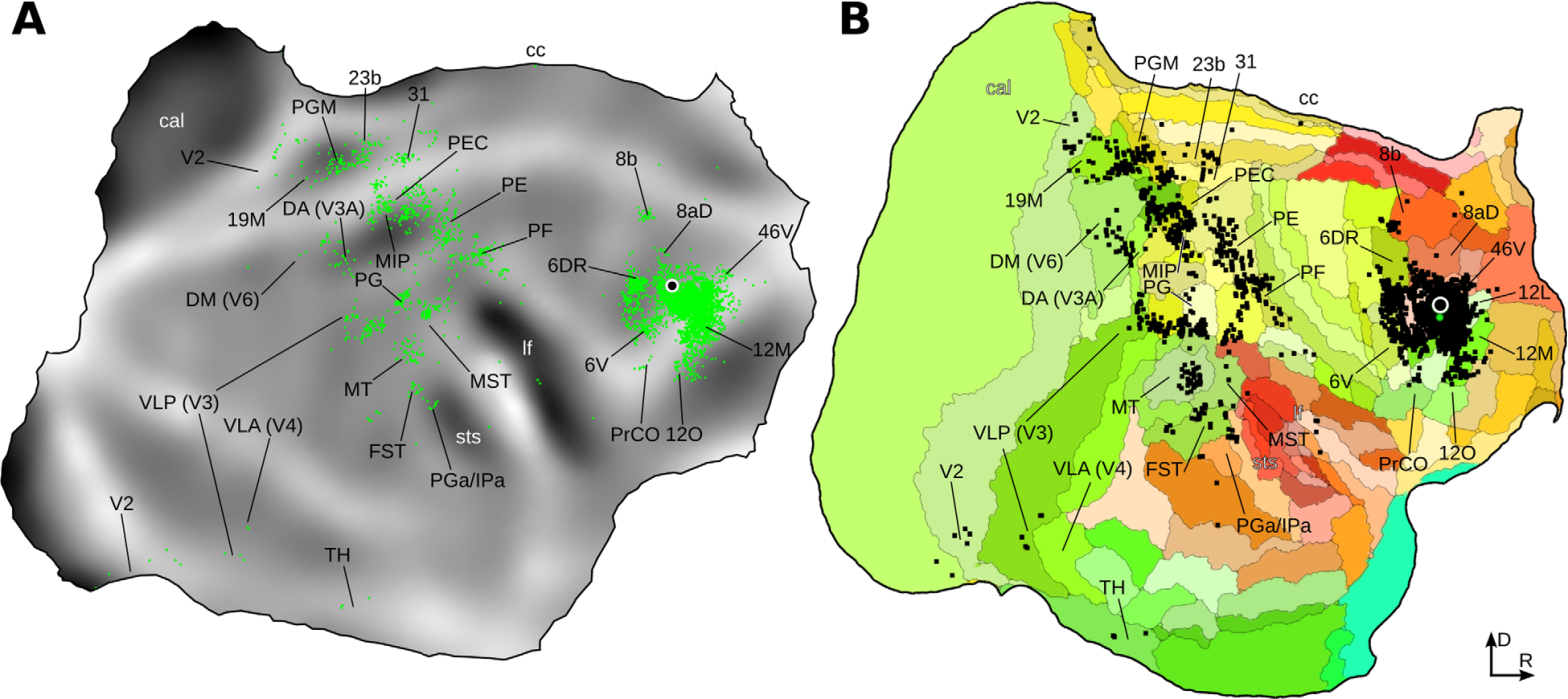
Comparison between the CJ108FE case (area 8aV injection) flat maps obtained manually, and by using the automated approach. **A**: Flat map obtained from a single individual case, published previously (Reser et al., 2013). Green points indicate individual cells. **B**: Cells from the same injection mapped into the reference brain, and projected onto the template flat map. Colors denote different cortical areas, while black points indicate individual cells. Several cortical areas have been labeled in both maps, using the convention in the original article. In both automated and manual approaches, similar projection patterns can be observed. [Color figure can be viewed in the online issue, which is available at wileyonlinelibrary.com.]

We illustrate in Figure 9 a direct comparison between flat map reconstructions from one case, prepared using the software CARET, obtained through manual (Reser et al., 2013) and automated procedures. The high level of concordance between the locations, shapes, and sizes of labeled cell patches is clear; thus, discrepancies in cell counts derive primarily from the exact placement of the lines that delineate the different areas. When one considers that the visual delineation of areas and individualized preparation of the summary in the first map required several months of work by highly trained experts (clearly, a rate‐limiting factor for large‐scale neuroanatomical mapping projects), and the inherent imprecision of the manual method, which is difficult to quantify precisely, it can be argued that the automated procedure results in an adequate, representative summary of the cortical connectivity of the injected site (in this case, a subdivision of the marmoset frontal eye field), which is achievable in a fraction of the processing time. Moreover, the automated procedure introduces the possibility of comparing data from many cases, in the same reference space, something that is impossible to do quantitatively with the manual approach. To illustrate this point, we provide as Supplementary Materials (S3_visualizations_of_injections.pdf) summary visualizations of the data from all 17 injections used in the present study, encompassing data from nine marmosets collected over several years.

The present workflow allows for estimate of the relative depth of labeled neurons in the cortex, but this needs to be acknowledged as an initial step towards a more accurate system that can allow assignment of laminar position. The thickness and relative depth of layers varies between cortical areas, as well as the degree of curvature of specific parts of the cortex (Hilgetag and Barbas, 2006). The current approach does not account for these factors. The ultimate solution will probably required automated or semiautomated detection of the level of layer 4 in different areas, as well as computational strategies to incorporate variations due to convexity or concavity (Waehnert et al., 2014; Leprince et al., 2015). It should be noted, however, that the current procedure provides accurate estimates of relative depth of labeled neurons, due to the fact that the surface of the cortex and the interface between cortex and white matter are prominent features, which constrain the registration procedure along the radial dimension of the cortex. Moreover, most of the marmoset cortex is relatively flat, thus mitigating the issue of curvature in comparison with other species such as the macaque.

### Limiting factors and proposed future developments

One of the limiting factors in the precision of the current procedure is the histological processing, which in part reflects the fact that the materials used were generated without consideration of the requirements for digital volume‐based reconstruction. Unless specific procedures are adopted to minimize artifacts, series of stained sections collected from free‐floating storage medium can suffer from various distortions such as tearing, shearing, and displacement of individual parts of the sections, and nonuniform shrinkage (Dauguet et al., 2007a). The obvious answer here is the adoption of systematic approaches for high‐quality and high‐throughput sectioning and section collection (e.g., Nissanov et al., 2001; Guy et al., 2015; Pinskiy et al., 2015), which can minimize distortions.

The template brain used in this study was derived from the electronic version of a printed atlas (Paxinos et al., 2012). This template was chosen as, at the moment of beginning the study, it was the only one satisfying the following conditions: 1) used a precise stereotaxic coordinate system; 2) comprised sufficiently dense sampling, which allowed 3D reconstruction with relatively modest interpolation; 3) comprised extensive delineation of the cerebral cortex; and 4) has already been used in many studies, allowing comparisons between automated and manual procedures in the same cases. Other available atlases of marmoset brain satisfy some, but not all, of the above conditions (Stephan, 1980; Newman et al., 2009; Yuasa et al., 2010; Hardman and Ashwell, 2012). Even though this is the best available template for the present purpose, it is still not optimal, for a few reasons. First, the distance between consecutive Nissl plates was 500 μm, which may have negatively influenced the registration in parts of the brain that changed rapidly from one section to another. In addition, cells located in regions consisting of numerous small areas were more prone to misassignment to an adjacent area due to this factor, since in many cases the procedure forced a binary decision between areas located in similar mediolateral and dorsoventral coordinates, in consecutive plates.

Additionally, the atlas is based on a single brain (female of age 3 years and 2 months and weight of 500 g, which was studied in great detail), and thus it does not account for anatomical variability of the adult marmoset brains. This biases the normalization process in such a way that brains that are more similar to the brain used to create the atlas coregister better, when compared with brains with different morphology (e.g., different length of the lateral sulcus). Although this problem was partially mitigated by introducing the landmarks, it remained one of the most significant limiting factors. Finally, because of a discrete parcellation (only a single cortical area could be assigned to given voxel) the template did not allow to account for smooth or unclear transitions between areas, which, as discussed above, is a real feature of primate association areas. For instance, in our experience the borders between dorsal prefrontal areas 8b, 9, and 10, visualized in coronal sections, are subject to greater uncertainty than those between subdivisions of area 8a, as assessed by comparisons between delineations by different experts.

The aforementioned problems suggest a need for probabilistic, population‐based marmoset brain atlases. Ideally, such templates need to incorporate data from many specimens to account for anatomical variability, as suggested for both human and nonhuman primates (e.g., Frey et al., 2011; Evans et al., 2012). Not less important, they should combine the histological data and data from noninvasive imaging techniques (MRI, DTI) to allow for finer neuroanatomical delineation or connectivity studies (see Annese, 2012). For example, areas that are highly myelinated relative to its neighbors, can be determined for each individual using MRI techniques, allowing for finer adjustment using additional landmarks.

Thanks to increasing popularity of the marmoset as a laboratory animal model, the problem of adequate marmoset brain atlases and templates is already being addressed. Some of these are targeted more towards functional imaging studies (e.g., Hikishima et al., 2011) while others attempt to combine imaging techniques with traditional histology (e.g., Hashikawa et al., 2015). Using a population‐based brain atlas as a template may increase the normalization accuracy in the present technique, and make further quantitative analyses, particularly with respect to the margin of error of the procedure, more reliable.

## SUMMARY AND CONCLUSION

We have presented a workflow for integration results of studies involving fluorescent tracer injections in the marmoset monkey brain. This was realized by 3D reconstruction and further spatial normalization of histological and fluorescent sections into a reference template. The described computational pipeline is based on commonly used open‐source components, and can be applied to data collected using traditional neuroanatomical procedures, as well as to data obtained with the requirements of the digital processing pipeline in mind. Thus, it may represent a way to reduce the use of animals and other resources towards the aim of obtaining a comprehensive and quantitative atlas of cortical connections.

In principle, the spatial normalization of the data into reference stereotaxic coordinates makes it feasible to compare the locations of labeled neurons to any parcellation scheme of the brain, to account for likely refinements prompted by future studies, or to completely discard parcellation attempts, and to conduct purely spatially based analyses. Moreover, it makes it possible to compare the results obtained using tracing techniques with data obtained using 3D functional and structural imaging methods.

Presently, the correlation between cell count obtained by the automated approach in comparison with the manual counts reported in earlier studies amounted to 0.83, while the spatial accuracy of the coregistration is at a level of 0.6 mm. However, we identify these current estimates as achievable minima, and suggest future developments that can improve precision. The proposed workflow is not envisioned as a direct replacement for traditional methods of studying and analyzing cortical connectivity patterns, based on carefully assigning individual cells to areas using detailed histological inspection. Despite the known imprecisions associated with the subjectivity of this approach, we believe that it will continue to represent the gold standard in neuroanatomy for the foreseeable future, pending the development of automated procedures for large‐scale automated histological parcellation. However, there are clear advantages in adopting a computational pipeline similar to the one described here, not only in terms of allowing faster throughput, but also in enabling efficient sharing of entire datasets, as opposed to selected illustrations of sections in print, and more sophisticated spatially based analyses of patterns of connectivity using multiple cases.

Finally, the present analyses directly quantify the likely margins of error involved in the registration of connectional data to a common template of the cerebral cortex. This information may be found useful in the context of allowing informed interpretation of data obtained in other projects that incorporate this approach, including various human brain mapping initiatives. Recognizing and being able to quantify the margin of error in assignment of cells and injection sites to particular structures of the brain is an important advance relative to traditional (publication‐based) ways of reporting neuroanatomical data.

## CONFLICT OF INTEREST

The authors declare no competing financial interests.

## ROLE OF AUTHORS

All authors had full access to all the data in the study and take responsibility for the integrity of the data and the accuracy of the data analysis. Study concept and design: PM, MGPR, PPM. Acquisition of data: PM, TAC, HHY, AT, MGPR. Programming: PM. Analysis and interpretation of data: PM, DKW, MGPR. Drafting of the article: PM, MGPR. Critical revision of the article for important intellectual content: PPM, DKW. Statistical analysis: PM. Obtained funding: DKW, MGPR, PPM.

## Supporting information

Supporting Information

Supporting Information

Supporting Information

Supporting Information
