# Supplementary material for: Towards a comprehensive atlas of cortical connections in a primate brain: Mapping tracer injection studies of the common marmoset into a reference digital template
Source: J Comp Neurol. 2016 Jun 3;524(11):2161–81. doi: 10.1002/cne.24023 (PMC4892968; doi:10.1002/cne.24023)
Supplement: Supplementary file 1 — Supporting Information [file CNE-524-2161-s001.pdf]

Reconstruction and coregistration parameters

		CJ70	CJ71
**Affine reconstruction step**			
General	Number of 3D + 2D alignment iterations	12	8
3D – 3D affine alignment	Similarity metric,<br>Similarity metric parameter:	MI / 32	MI / 32
	Number of affine iterations	10000x10000x10000x10000x10000	10000x10000x10000x10000x10000
	Affine transformation type	Affine	Affine
	Histogram matching	Yes	Yes
2D – 2D affine alignment	Similarity metric,<br>Similarity metric parameter:	MI / 32	MI / 32
	Number of affine iterations	10000x10000x10000x10000x10000	10000x10000x10000x10000x10000
	Affine transformation type	Rigid	Rigid
	Histogram matching	Yes	Yes
**Deformable reconstruction step**			
	Number of iterations<br>Of the deformable coregistration step	8	8
	Similarity metric,<br>Similarity metric parameter:	CC / 4	CC / 4
	Deformable transformation model,<br>Gradient step length:	SyN, 0.01	SyN, 0.01
	Regularization:<br>Gradient sigma,<br>Deformation field sigma:	Gauss, 2.0,1.0	Gauss, 2.0,1.0
	Deformable coregistration iterations	1000x1000x1000x1000x1000x0	1000x1000x1000x1000x1000x0
**3D – 3D coregistration step**			
Affine alignment	Similarity metric,<br>Similarity metric parameter:	MI / 32	MI / 32
	Number of affine iterations	10000x10000x10000x10000x10000	10000x10000x10000x10000x10000
Deformable warping	Similarity metric,<br>Similarity metric parameter:	CC / 4	CC / 4
	CC weight	0.5	0.5
	PSE weight	0.5	0.5
	Deformable transformation model,<br>Gradient step length:	SyN, 0.25	SyN, 0.25
	Deformable iterations	1000x1000x1000x1000x1000x100	1000x1000x1000x1000x1000x100
	Regularization:<br>Gradient sigma,<br>Deformation field sigma:	Gauss, 1.0,0.0	Gauss, 1.0,0.0

**CJ73**	**CJ74**	**CJ75**	**CJ83**	**CJ94**
12	12	10	8	10
MI / 32<br>10000x10000x10000x10000x10000<br>Affine<br>Yes	MI / 32<br>10000x10000x10000x10000x10000<br>Affine<br>Yes	MI / 32<br>10000x10000x10000x10000x10000<br>Affine<br>Yes	MI / 32<br>10000x10000x10000x10000x10000<br>Affine<br>Yes	MI / 32<br>10000x10000x10000x10000x10000<br>Affine<br>Yes
MI / 32<br>10000x10000x10000x10000x10000<br>Rigid<br>Yes	MI / 32<br>10000x10000x10000x10000x10000<br>Rigid<br>Yes	MI / 32<br>10000x10000x10000x10000x10000<br>Rigid<br>Yes	MI / 32<br>10000x10000x10000x10000x10000<br>Rigid<br>Yes	MI / 32<br>10000x10000x10000x10000x10000<br>Rigid<br>Yes
8<br>CC / 4<br>SyN, 0.01<br>Gauss, 2.0,1.0<br>1000x1000x1000x1000x1000x0	8<br>CC / 4<br>SyN, 0.01<br>Gauss, 2.0,1.0<br>1000x1000x1000x1000x1000x0	8<br>CC / 4<br>SyN, 0.01<br>Gauss, 2.0,1.0<br>1000x1000x1000x1000x1000x0	8<br>CC / 4<br>SyN, 0.01<br>Gauss, 2.0,1.0<br>1000x1000x1000x1000x1000x0	8<br>CC / 4<br>SyN, 0.01<br>Gauss, 2.0,1.0<br>1000x1000x1000x1000x1000x0
MI / 32<br>10000x10000x10000x10000x10000	MI / 32<br>10000x10000x10000x10000x10000	MI / 32<br>10000x10000x10000x10000x10000	MI / 32<br>10000x10000x10000x10000x10000	MI / 32<br>10000x10000x10000x10000x10000
CC / 4<br>0.5<br>0.5<br>SyN, 0.25<br>1000x1000x1000x1000x1000x100<br>Gauss, 1.0,0.0	CC / 4<br>0.5<br>0.5<br>SyN, 0.25<br>1000x1000x1000x1000x1000x100<br>Gauss, 1.0,0.0	CC / 4<br>0.5<br>0.5<br>SyN, 0.25<br>1000x1000x1000x1000x1000x100<br>Gauss, 1.0,0.0	CC / 4<br>0.5<br>0.5<br>SyN, 0.25<br>1000x1000x1000x1000x1000x100<br>Gauss, 1.0,0.0	CC / 4<br>0.5<br>0.5<br>SyN, 0.25<br>1000x1000x1000x1000x1000x100<br>Gauss, 1.0,0.0

CJ108	CJ125
10	10
MI / 32<br>10000x10000x10000x10000x10000<br>Affine<br>Yes	MI / 32<br>10000x10000x10000x10000x10000<br>Affine<br>Yes
MI / 32<br>10000x10000x10000x10000x10000<br>Rigid<br>Yes	MI / 32<br>10000x10000x10000x10000x10000<br>Rigid<br>Yes
12<br>CC / 4<br>SyN, 0.01<br>Gauss, 2.0,1.0<br>1000x1000x1000x1000x1000x0	10<br>CC / 4<br>SyN, 0.01<br>Gauss, 2.0,1.0<br>1000x1000x1000x1000x1000x0
MI / 32<br>10000x10000x10000x10000x10000	MI / 32<br>10000x10000x10000x10000x10000
CC / 4<br>0.5<br>0.5<br>SyN, 0.25<br>1000x1000x1000x1000x1000x100<br>Gauss, 1.0,0.0	CC / 4<br>0.5<br>0.5<br>SyN, 0.25<br>1000x1000x1000x1000x1000x100<br>Gauss, 1.0,0.0
