# Supplementary material for: Towards a comprehensive atlas of cortical connections in a primate brain: Mapping tracer injection studies of the common marmoset into a reference digital template
Source: J Comp Neurol. 2016 Jun 3;524(11):2161–81. doi: 10.1002/cne.24023 (PMC4892968; doi:10.1002/cne.24023)
Supplement: Supplementary file 2 — Supporting Information [file CNE-524-2161-s002.pdf]

Cells mapping accuracy asseseme

Total number of points:	**546**
Pearson's r	**0.83**

Total number of points which is exactly zero in the reference data:	**229**
Of which 177 is exactly zero as established by the mapping	**177**
	**77.3%**

Total number of points which is less or euqal to 0.05 in the reference data:	**277**
Analogous number based on the mapping:	**229**
	**82.7%**

How many points have the reference percentage more than 0.1%?	**209**
Which is 38.3% of the total number of points.	**546**
	**38.3%**

How many points have the reference percentage more than 2%?	**84**
	**546**
	**15.4%**

Total nu	546		
	Should be zero	and is Zero	
	229	177	77.3%
Total points / zero points:			
	546	229	41.9%
	Should be less or	and is <= 0.05	
	277	229	82.7%
Total points / less <0.05 points:			
	546	277	50.7%

How many points are shown on the plot: both has to be	209
How many points are shown on the plot: both has to be	84

Complete list of the results

**Case – Tracer – list of aggregated cortical areas**	**Reference cell percentage As in the original articles (list the articles here)**	**Cell percentage calculated Basing on the proposed Reconstruction and normalization approach**	Should be Zero And is zero	Should Be Zero	Should be less or Equal 0.05%	and is <= 0.05	Should be (and is) => 0.1%	Should be (and is) => 2%
CJ74-FB-A9	12.7	6.80	0	0	0	0	1	1
CJ74-FB-A10	3.3	0.00	0	0	0	0	0	0
CJ74-FB-A46D;A46V	14.8	0.71	0	0	0	0	1	0
CJ74-FB-A8b	0	0.00	1	1	1	1	0	0
CJ74-FB-A8aD	1.8	13.46	0	0	0	0	1	1
CJ74-FB-A8aV	0.3	0.42	0	0	0	0	1	0
CJ74-FB-A47L(12L)	1.8	0.85	0	0	0	0	1	0
CJ74-FB-A47M(12M)	1.3	0.14	0	0	0	0	1	0
CJ74-FB-A47O(12O)	0.3	1.98	0	0	0	0	1	0
CJ74-FB-A45	0	0.00	1	1	1	1	0	0
CJ74-FB-ProM(PrCO)	0	0.00	1	1	1	1	0	0
CJ74-FB-A11	0.2	0.28	0	0	0	0	1	0
CJ74-FB-A13a;A13b	0	0.00	1	1	1	1	0	0
CJ74-FB-A13L	1.8	0.00	0	0	0	0	0	0
CJ74-FB-A13M	0	0.42	0	1	1	0	0	0
CJ74-FB-Gu	0	0.00	1	1	1	1	0	0
CJ74-FB-OPAl	0	1.84	0	1	1	0	0	0
CJ74-FB-OPro	0	0.14	0	1	1	0	0	0
CJ74-FB-A14R;A14C	0.2	0.14	0	0	0	0	1	0
CJ74-FB-A32	16.9	25.92	0	0	0	0	1	1
CJ74-FB-A32V	0	0.00	1	1	1	1	0	0
CJ74-FB-A4ab;A4c	0	0.00	1	1	1	1	0	0
CJ74-FB-A6DC	0	0.14	0	1	1	0	0	0
CJ74-FB-A6DR	4.8	6.09	0	0	0	0	1	1
CJ74-FB-A6M	0	0.14	0	1	1	0	0	0
CJ74-FB-A6Va;A6Vb	0	0.00	1	1	1	1	0	0
CJ74-FB-A8C	0	0.00	1	1	1	1	0	0
CJ74-FB-A24b	0.8	3.12	0	0	0	0	1	1
CJ74-FB-A24c;A24d	0.2	2.41	0	0	0	0	1	1
CJ74-FB-A3a	0.2	0.14	0	0	0	0	1	0
CJ74-FB-A1/2	0	0.00	1	1	1	1	0	0
CJ74-FB-S2E;S2I;S2PV;S2PR	0	0.00	1	1	1	1	0	0
CJ74-FB-AI;DI;GI;IPro;PaIL;PaIM;ReI;TPro	0	0.00	1	1	1	1	0	0
CJ74-FB-AuA1;AuAL;AuCL;AuCM;AuML;AuR;AuRM;AuRTL;AuRTM;AuRT	0	0.14	0	1	1	0	0	0
CJ74-FB-AuRPB;AuCPB	1	0.14	0	0	0	0	1	0
CJ74-FB-TPt	0	0.00	1	1	1	1	0	0
CJ74-FB-STR	0	0.00	1	1	1	1	0	0
CJ74-FB-TE1	0	0.00	1	1	1	1	0	0
CJ74-FB-TE2	0	0.00	1	1	1	1	0	0
CJ74-FB-TE3	0.3	0.28	0	0	0	0	1	0
CJ74-FB-TEO	0	0.00	1	1	1	1	0	0
CJ74-FB-TPO(STP)	2.5	2.41	0	0	0	0	1	1
CJ74-FB-PGa/IPa(FSTv)	0.7	0.71	0	0	0	0	1	0
CJ74-FB-A36	0.4	0.14	0	0	0	0	1	0
CJ74-FB-TPPro	0.3	0.28	0	0	0	0	1	0
CJ74-FB-Ent	0	0.00	1	1	1	1	0	0
CJ74-FB-TF;TL	0	0.00	1	1	1	1	0	0
CJ74-FB-TH	0	0.00	1	1	1	1	0	0
CJ74-FB-TFO;TLO	0	0.00	1	1	1	1	0	0
CJ74-FB-AIP	0	0.00	1	1	1	1	0	0
CJ74-FB-LIP	0	0.00	1	1	1	1	0	0
CJ74-FB-MIP	0	0.00	1	1	1	1	0	0
CJ74-FB-VIP	0	0.00	1	1	1	1	0	0
CJ74-FB-PE;PEC	0	0.00	1	1	1	1	0	0
CJ74-FB-PF	0	0.00	1	1	1	1	0	0
CJ74-FB-PFG	0.05	0.00	0	0	1	1	0	0
CJ74-FB-PG	0	0.00	1	1	1	1	0	0
CJ74-FB-OPt	0	0.00	1	1	1	1	0	0
CJ74-FB-PGM	0.5	0.71	0	0	0	0	1	0
CJ74-FB-A31	0	0.00	1	1	1	1	0	0
CJ74-FB-A29a-c;A29d	2.8	1.42	0	0	0	0	1	0
CJ74-FB-A30	7.9	5.52	0	0	0	0	1	1
CJ74-FB-A23a	13.9	11.90	0	0	0	0	1	1
CJ74-FB-A23b;A23c	0.8	0.99	0	0	0	0	1	0
CJ74-FB-A23V	0.5	1.42	0	0	0	0	1	0
CJ74-FB-ProSt	3.1	1.70	0	0	0	0	1	0
CJ74-FB-V2	0	0.00	1	1	1	1	0	0
CJ74-FB-V3(VLP)	0	0.00	1	1	1	1	0	0
CJ74-FB-V4(VLA)	0	0.00	1	1	1	1	0	0
CJ74-FB-V5(MT)	0	0.00	1	1	1	1	0	0
CJ74-FB-V4T(MTC)	0	0.00	1	1	1	1	0	0
CJ74-FB-MST	0	0.00	1	1	1	1	0	0
CJ74-FB-FST	0	0.28	0	1	1	0	0	0
CJ74-FB-V6(DM)	0	0.00	1	1	1	1	0	0
CJ74-FB-V3A(DA)	0	0.00	1	1	1	1	0	0
CJ74-FB-A19DI	0	0.00	1	1	1	1	0	0
CJ74-FB-V6A(PPM)	0	0.00	1	1	1	1	0	0
CJ74-FB-A19M	0	0.42	0	1	1	0	0	0
CJ83-DY-A9	22.6	8.14	0	0	0	0	1	1

CJ83-DY-A10	8.2	2.20	0	0	0	0	1	1
CJ83-DY-A46D;A46V	1.4	4.90	0	0	0	0	1	1
CJ83-DY-A8b	0	0.00	1	1	1	1	0	0
CJ83-DY-A8aD	21.1	28.76	0	0	0	0	1	1
CJ83-DY-A8aV	1.1	0.78	0	0	0	0	1	0
CJ83-DY-A47L(12L)	0.7	0.49	0	0	0	0	1	0
CJ83-DY-A47M(12M)	0.9	0.73	0	0	0	0	1	0
CJ83-DY-A47O(12O)	1.5	1.17	0	0	0	0	1	0
CJ83-DY-A45	0	0.01	0	1	1	1	0	0
CJ83-DY-ProM(PrCO)	0	0.01	0	1	1	1	0	0
CJ83-DY-A11	0.8	0.47	0	0	0	0	1	0
CJ83-DY-A13a;A13b	0	0.00	1	1	1	1	0	0
CJ83-DY-A13L	0.05	0.20	0	0	1	0	0	0
CJ83-DY-A13M	0.2	0.10	0	0	0	0	1	0
CJ83-DY-Gu	0.05	0.00	0	0	1	1	0	0
CJ83-DY-OPAl	0.2	0.06	0	0	0	0	0	0
CJ83-DY-OPro	0.1	0.01	0	0	0	0	0	0
CJ83-DY-A14R;A14C	0.2	0.02	0	0	0	0	0	0
CJ83-DY-A32	7.9	10.40	0	0	0	0	1	1
CJ83-DY-A32V	0.1	0.01	0	0	0	0	0	0
CJ83-DY-A4ab;A4c	0	0.09	0	1	1	0	0	0
CJ83-DY-A6DC	4.8	0.21	0	0	0	0	1	0
CJ83-DY-A6DR	11.5	12.07	0	0	0	0	1	1
CJ83-DY-A6M	1.4	7.57	0	0	0	0	1	1
CJ83-DY-A6Va;A6Vb	0.05	0.01	0	0	1	1	0	0
CJ83-DY-A8C	0	0.00	1	1	1	1	0	0
CJ83-DY-A24b	1.3	3.28	0	0	0	0	1	1
CJ83-DY-A24c;A24d	0.1	5.74	0	0	0	0	1	1
CJ83-DY-A3a	0	0.00	1	1	1	1	0	0
CJ83-DY-A1/2	0	0.00	1	1	1	1	0	0
CJ83-DY-S2E;S2I;S2PV;S2PR	0	0.00	1	1	1	1	0	0
CJ83-DY-AI;DI;GI;IPro;PaIL;PaIM;ReI;TPro	0.05	0.01	0	0	1	1	0	0
CJ83-DY-AuA1;AuAL;AuCL;AuCM;AuML;AuR;AuRM;AuRTL;AuRTM;AuRT	0	0.47	0	1	1	0	0	0
CJ83-DY-AuRPB;AuCPB	0.2	1.31	0	0	0	0	1	0
CJ83-DY-TPt	0	0.01	0	1	1	1	0	0
CJ83-DY-STR	0.1	0.19	0	0	0	0	1	0
CJ83-DY-TE1	0.1	0.01	0	0	0	0	0	0
CJ83-DY-TE2	0.05	0.02	0	0	1	1	0	0
CJ83-DY-TE3	0.2	0.02	0	0	0	0	0	0
CJ83-DY-TEO	0	0.00	1	1	1	1	0	0
CJ83-DY-TPO(STP)	2.4	1.35	0	0	0	0	1	0
CJ83-DY-PGa/IPa(FSTv)	0.4	0.00	0	0	0	0	0	0
CJ83-DY-A36	0.05	0.01	0	0	1	1	0	0
CJ83-DY-TPPro	0.05	0.01	0	0	1	1	0	0
CJ83-DY-Ent	0.05	0.06	0	0	1	0	0	0
CJ83-DY-TF;TL	0.1	0.00	0	0	0	0	0	0
CJ83-DY-TH	0.1	0.16	0	0	0	0	1	0
CJ83-DY-TFO;TLO	0	0.01	0	1	1	1	0	0
CJ83-DY-AIP	0	0.06	0	1	1	0	0	0
CJ83-DY-LIP	0	0.01	0	1	1	1	0	0
CJ83-DY-MIP	0	0.01	0	1	1	1	0	0
CJ83-DY-VIP	0	0.00	1	1	1	1	0	0
CJ83-DY-PE;PEC	0	0.00	1	1	1	1	0	0
CJ83-DY-PF	0	0.00	1	1	1	1	0	0
CJ83-DY-PFG	0.1	0.05	0	0	0	0	0	0
CJ83-DY-PG	0	0.01	0	1	1	1	0	0
CJ83-DY-OPt	0	0.00	1	1	1	1	0	0
CJ83-DY-PGM	0.05	0.10	0	0	1	0	0	0
CJ83-DY-A31	0.05	0.01	0	0	1	1	0	0
CJ83-DY-A29a-c;A29d	2.1	0.71	0	0	0	0	1	0
CJ83-DY-A30	2.4	3.24	0	0	0	0	1	1
CJ83-DY-A23a	1.1	1.64	0	0	0	0	1	0
CJ83-DY-A23b;A23c	0.6	0.40	0	0	0	0	1	0
CJ83-DY-A23V	0.1	0.09	0	0	0	0	0	0
CJ83-DY-ProSt	0.05	0.14	0	0	1	0	0	0
CJ83-DY-V2	0	0.02	0	1	1	1	0	0
CJ83-DY-V3(VLP)	0	0.00	1	1	1	1	0	0
CJ83-DY-V4(VLA)	0.05	0.00	0	0	1	1	0	0
CJ83-DY-V5(MT)	0	0.00	1	1	1	1	0	0
CJ83-DY-V4T(MTC)	0	0.00	1	1	1	1	0	0
CJ83-DY-MST	0	0.03	0	1	1	1	0	0
CJ83-DY-FST	0.05	0.01	0	0	1	1	0	0
CJ83-DY-V6(DM)	0	0.00	1	1	1	1	0	0
CJ83-DY-V3A(DA)	0	0.00	1	1	1	1	0	0
CJ83-DY-A19DI	0	0.00	1	1	1	1	0	0
CJ83-DY-V6A(PPM)	0	0.00	1	1	1	1	0	0
CJ83-DY-A19M	0	0.00	1	1	1	1	0	0
CJ70-FR-A9	15	0.26	0	0	0	0	1	0
CJ70-FR-A10	2.5	10.24	0	0	0	0	1	1
CJ70-FR-A46D;A46V	7.1	16.27	0	0	0	0	1	1
CJ70-FR-A8b	8.2	3.67	0	0	0	0	1	1
CJ70-FR-A8aD	0	0.00	1	1	1	1	0	0
CJ70-FR-A8aV	1.1	8.66	0	0	0	0	1	1
CJ70-FR-A47L(12L)	4	6.04	0	0	0	0	1	1
CJ70-FR-A47M(12M)	0.8	2.36	0	0	0	0	1	1
CJ70-FR-A47O(12O)	0	0.00	1	1	1	1	0	0
CJ70-FR-A45	0	0.00	1	1	1	1	0	0
CJ70-FR-ProM(PrCO)	0	0.00	1	1	1	1	0	0
CJ70-FR-A11	4	2.62	0	0	0	0	1	1
CJ70-FR-A13a;A13b	0	0.00	1	1	1	1	0	0
CJ70-FR-A13L	2.8	0.00	0	0	0	0	0	0
CJ70-FR-A13M	0.6	0.52	0	0	0	0	1	0
CJ70-FR-Gu	0	0.00	1	1	1	1	0	0
CJ70-FR-OPAl	0	0.00	1	1	1	1	0	0
CJ70-FR-OPro	0	0.00	1	1	1	1	0	0
CJ70-FR-A14R;A14C	0	0.00	1	1	1	1	0	0
CJ70-FR-A32	0	0.00	1	1	1	1	0	0
CJ70-FR-A32V	0	0.00	1	1	1	1	0	0
CJ70-FR-A4ab;A4c	0	0.00	1	1	1	1	0	0
CJ70-FR-A6DC	0	0.00	1	1	1	1	0	0
CJ70-FR-A6DR	6.8	3.15	0	0	0	0	1	1
CJ70-FR-A6M	0.8	0.79	0	0	0	0	1	0
CJ70-FR-A6Va;A6Vb	0.8	1.57	0	0	0	0	1	0
CJ70-FR-A8C	0	0.00	1	1	1	1	0	0
CJ70-FR-A24b	1.7	0.00	0	0	0	0	0	0
CJ70-FR-A24c;A24d	0	0.26	0	1	1	0	0	0
CJ70-FR-A3a	0	0.00	1	1	1	1	0	0
CJ70-FR-A1/2	0	0.00	1	1	1	1	0	0
CJ70-FR-S2E;S2I;S2PV;S2PR	2	3.41	0	0	0	0	1	1
CJ70-FR-AI;DI;GI;IPro;PaIL;PaIM;ReI;TPro	1.4	0.52	0	0	0	0	1	0
CJ70-FR-AuA1;AuAL;AuCL;AuCM;AuML;AuR;AuRM;AuRTL;AuRTM;AuRT	0	1.57	0	1	1	0	0	0
CJ70-FR-AuRPB;AuCPB	0.3	0.00	0	0	0	0	0	0

CJ70-FR-TPt	4.5	0.00	0	0	0	0	0	0
CJ70-FR-STR	0	0.00	1	1	1	1	0	0
CJ70-FR-TE1	0	0.00	1	1	1	1	0	0
CJ70-FR-TE2	0	0.00	1	1	1	1	0	0
CJ70-FR-TE3	0	0.00	1	1	1	1	0	0
CJ70-FR-TEO	0	0.00	1	1	1	1	0	0
CJ70-FR-TPO(STP)	1.1	0.79	0	0	0	0	1	0
CJ70-FR-PGa/IPa(FSTv)	0.8	0.26	0	0	0	0	1	0
CJ70-FR-A36	0	0.00	1	1	1	1	0	0
CJ70-FR-TPPro	0	0.00	1	1	1	1	0	0
CJ70-FR-Ent	0	0.00	1	1	1	1	0	0
CJ70-FR-TF;TL	0	0.00	1	1	1	1	0	0
CJ70-FR-TH	0	0.26	0	1	1	0	0	0
CJ70-FR-TFO;TLO	0.3	0.00	0	0	0	0	0	0
CJ70-FR-AIP	0	0.00	1	1	1	1	0	0
CJ70-FR-LIP	0.1	0.52	0	0	0	0	1	0
CJ70-FR-MIP	0	0.00	1	1	1	1	0	0
CJ70-FR-VIP	0	0.00	1	1	1	1	0	0
CJ70-FR-PE;PEC	0.6	3.41	0	0	0	0	1	1
CJ70-FR-PF	2.8	3.67	0	0	0	0	1	1
CJ70-FR-PFG	0	2.89	0	1	1	0	0	1
CJ70-FR-PG	0.5	0.00	0	0	0	0	0	0
CJ70-FR-OPt	0	0.00	1	1	1	1	0	0
CJ70-FR-PGM	0.3	0.26	0	0	0	0	1	0
CJ70-FR-A31	0.3	1.05	0	0	0	0	1	0
CJ70-FR-A29a-c;A29d	0.6	0.52	0	0	0	0	1	0
CJ70-FR-A30	4	0.26	0	0	0	0	1	0
CJ70-FR-A23a	16.7	13.39	0	0	0	0	1	1
CJ70-FR-A23b;A23c	6.5	9.19	0	0	0	0	1	1
CJ70-FR-A23V	0	0.26	0	1	1	0	0	0
CJ70-FR-ProSt	0	0.00	1	1	1	1	0	0
CJ70-FR-V2	0	0.00	1	1	1	1	0	0
CJ70-FR-V3(VLP)	0	0.00	1	1	1	1	0	0
CJ70-FR-V4(VLA)	0	0.00	1	1	1	1	0	0
CJ70-FR-V5(MT)	0	0.00	1	1	1	1	0	0
CJ70-FR-V4T(MTC)	0	0.00	1	1	1	1	0	0
CJ70-FR-MST	0.8	0.52	0	0	0	0	1	0
CJ70-FR-FST	0.3	0.00	0	0	0	0	0	0
CJ70-FR-V6(DM)	0	0.00	1	1	1	1	0	0
CJ70-FR-V3A(DA)	0	0.00	1	1	1	1	0	0
CJ70-FR-A19DI	0	0.00	1	1	1	1	0	0
CJ70-FR-V6A(PPM)	0	0.26	0	1	1	0	0	0
CJ70-FR-A19M	0.3	0.26	0	0	0	0	1	0
CJ108-FR-A9	10.5	1.99	0	0	0	0	1	0
CJ108-FR-A10	1.1	1.77	0	0	0	0	1	0
CJ108-FR-A46D;A46V	5.2	8.09	0	0	0	0	1	1
CJ108-FR-A8b	10.5	11.41	0	0	0	0	1	1
CJ108-FR-A8aD	0	0.00	1	1	1	1	0	0
CJ108-FR-A8aV	4.6	5.08	0	0	0	0	1	1
CJ108-FR-A47L(12L)	6.6	4.93	0	0	0	0	1	1
CJ108-FR-A47M(12M)	0.2	1.47	0	0	0	0	1	0
CJ108-FR-A47O(12O)	0	0.00	1	1	1	1	0	0
CJ108-FR-A45	0	0.00	1	1	1	1	0	0
CJ108-FR-ProM(PrCO)	0	0.00	1	1	1	1	0	0
CJ108-FR-A11	0.9	3.16	0	0	0	0	1	1
CJ108-FR-A13a;A13b	0	0.00	1	1	1	1	0	0
CJ108-FR-A13L	3.2	1.03	0	0	0	0	1	0
CJ108-FR-A13M	0.5	0.22	0	0	0	0	1	0
CJ108-FR-Gu	0	0.00	1	1	1	1	0	0
CJ108-FR-OPAl	0	0.00	1	1	1	1	0	0
CJ108-FR-OPro	0	0.07	0	1	1	0	0	0
CJ108-FR-A14R;A14C	0	0.00	1	1	1	1	0	0
CJ108-FR-A32	0.1	0.00	0	0	0	0	0	0
CJ108-FR-A32V	0	0.00	1	1	1	1	0	0
CJ108-FR-A4ab;A4c	0	0.00	1	1	1	1	0	0
CJ108-FR-A6DC	0.7	0.00	0	0	0	0	0	0
CJ108-FR-A6DR	11.3	13.10	0	0	0	0	1	1
CJ108-FR-A6M	0	0.52	0	1	1	0	0	0
CJ108-FR-A6Va;A6Vb	0	0.00	1	1	1	1	0	0
CJ108-FR-A8C	0	0.66	0	1	1	0	0	0
CJ108-FR-A24b	0.2	0.15	0	0	0	0	1	0
CJ108-FR-A24c;A24d	0	0.22	0	1	1	0	0	0
CJ108-FR-A3a	0	0.00	1	1	1	1	0	0
CJ108-FR-A1/2	0	0.00	1	1	1	1	0	0
CJ108-FR-S2E;S2I;S2PV;S2PR	0.05	0.00	0	0	1	1	0	0
CJ108-FR-AI;DI;GI;IPro;PaIL;PaIM;ReI;TPro	0	0.07	0	1	1	0	0	0
CJ108-FR-AuA1;AuAL;AuCL;AuCM;AuML;AuR;AuRM;AuRTL;AuRTM;AuRT	0	0.15	0	1	1	0	0	0
CJ108-FR-AuRPB;AuCPB	0.1	0.00	0	0	0	0	0	0
CJ108-FR-TPt	0.7	0.29	0	0	0	0	1	0
CJ108-FR-STR	0	0.00	1	1	1	1	0	0
CJ108-FR-TE1	0	0.00	1	1	1	1	0	0
CJ108-FR-TE2	0	0.00	1	1	1	1	0	0
CJ108-FR-TE3	0	0.00	1	1	1	1	0	0
CJ108-FR-TEO	0	0.00	1	1	1	1	0	0
CJ108-FR-TPO(STP)	0.1	0.07	0	0	0	0	0	0
CJ108-FR-PGa/IPa(FSTv)	0	0.00	1	1	1	1	0	0
CJ108-FR-A36	0	0.00	1	1	1	1	0	0
CJ108-FR-TPPro	0	0.00	1	1	1	1	0	0
CJ108-FR-Ent	0	0.07	0	1	1	0	0	0
CJ108-FR-TF;TL	0	0.00	1	1	1	1	0	0
CJ108-FR-TH	0.1	0.00	0	0	0	0	0	0
CJ108-FR-TFO;TLO	0.1	0.07	0	0	0	0	0	0
CJ108-FR-AIP	0	0.00	1	1	1	1	0	0
CJ108-FR-LIP	0.5	0.96	0	0	0	0	1	0
CJ108-FR-MIP	0	0.15	0	1	1	0	0	0
CJ108-FR-VIP	0.2	0.00	0	0	0	0	0	0
CJ108-FR-PE;PEC	0.7	0.44	0	0	0	0	1	0
CJ108-FR-PF	0.8	0.00	0	0	0	0	0	0
CJ108-FR-PFG	0	1.25	0	1	1	0	0	0
CJ108-FR-PG	1.8	0.96	0	0	0	0	1	0
CJ108-FR-OPt	0	0.00	1	1	1	1	0	0
CJ108-FR-PGM	1.1	3.61	0	0	0	0	1	1
CJ108-FR-A31	0.9	1.55	0	0	0	0	1	0
CJ108-FR-A29a-c;A29d	5.9	7.14	0	0	0	0	1	1
CJ108-FR-A30	10.9	2.43	0	0	0	0	1	1
CJ108-FR-A23a	5.9	8.24	0	0	0	0	1	1
CJ108-FR-A23b;A23c	10.2	16.04	0	0	0	0	1	1
CJ108-FR-A23V	0.6	1.03	0	0	0	0	1	0
CJ108-FR-ProSt	0.2	0.00	0	0	0	0	0	0
CJ108-FR-V2	0	0.07	0	1	1	0	0	0
CJ108-FR-V3(VLP)	0	0.00	1	1	1	1	0	0
CJ108-FR-V4(VLA)	0	0.00	1	1	1	1	0	0

CJ108-FR-V5(MT)	0	0.00	1	1	1	1	0	0
CJ108-FR-V4T(MTC)	0	0.00	1	1	1	1	0	0
CJ108-FR-MST	0.2	0.00	0	0	0	0	0	0
CJ108-FR-FST	0	0.00	1	1	1	1	0	0
CJ108-FR-V6(DM)	0	0.00	1	1	1	1	0	0
CJ108-FR-V3A(DA)	0	0.00	1	1	1	1	0	0
CJ108-FR-A19DI	0	0.00	1	1	1	1	0	0
CJ108-FR-V6A(PPM)	0	0.07	0	1	1	0	0	0
CJ108-FR-A19M	0.2	1.18	0	0	0	0	1	0
CJ75-DY-A9	0	0.00	1	1	1	1	0	0
CJ75-DY-A10	0.2	0.07	0	0	0	0	0	0
CJ75-DY-A46D;A46V	3.4	0.14	0	0	0	0	1	0
CJ75-DY-A8b	0.1	0.07	0	0	0	0	0	0
CJ75-DY-A8aD	0.8	0.19	0	0	0	0	1	0
CJ75-DY-A8aV	0	0.00	1	1	1	1	0	0
CJ75-DY-A47L(12L)	36.9	23.06	0	0	0	0	1	1
CJ75-DY-A47M(12M)	0.8	2.24	0	0	0	0	1	1
CJ75-DY-A47O(12O)	0.2	0.26	0	0	0	0	1	0
CJ75-DY-A45	5.3	11.96	0	0	0	0	1	1
CJ75-DY-ProM(PrCO)	0.1	0.05	0	0	0	0	0	0
CJ75-DY-A11	0.1	0.01	0	0	0	0	0	0
CJ75-DY-A13a;A13b	0.1	0.00	0	0	0	0	0	0
CJ75-DY-A13L	0.2	0.18	0	0	0	0	1	0
CJ75-DY-A13M	0.05	0.03	0	0	1	1	0	0
CJ75-DY-Gu	0	0.01	0	1	1	1	0	0
CJ75-DY-OPAl	0	0.04	0	1	1	1	0	0
CJ75-DY-OPro	0	0.00	1	1	1	1	0	0
CJ75-DY-A14R;A14C	0.05	0.00	0	0	1	1	0	0
CJ75-DY-A32	0.05	0.00	0	0	1	1	0	0
CJ75-DY-A32V	0	0.00	1	1	1	1	0	0
CJ75-DY-A4ab;A4c	0.05	0.03	0	0	1	1	0	0
CJ75-DY-A6DC	0.5	0.45	0	0	0	0	1	0
CJ75-DY-A6DR	4.3	1.10	0	0	0	0	1	0
CJ75-DY-A6M	0.05	0.07	0	0	1	0	0	0
CJ75-DY-A6Va;A6Vb	2.5	2.94	0	0	0	0	1	1
CJ75-DY-A8C	0.8	2.35	0	0	0	0	1	1
CJ75-DY-A24b	0.05	0.01	0	0	1	1	0	0
CJ75-DY-A24c;A24d	0	0.00	1	1	1	1	0	0
CJ75-DY-A3a	0.05	0.01	0	0	1	1	0	0
CJ75-DY-A1/2	0.05	0.00	0	0	1	1	0	0
CJ75-DY-S2E;S2I;S2PV;S2PR	0.05	0.00	0	0	1	1	0	0
CJ75-DY-AI;DI;GI;IPro;PaIL;PaIM;ReI;TPro	0.2	0.01	0	0	0	0	0	0
CJ75-DY-AuA1;AuAL;AuCL;AuCM;AuML;AuR;AuRM;AuRTL;AuRTM;AuRT	0	0.10	0	1	1	0	0	0
CJ75-DY-AuRPB;AuCPB	0.1	0.00	0	0	0	0	0	0
CJ75-DY-TPt	0	0.00	1	1	1	1	0	0
CJ75-DY-STR	0	0.00	1	1	1	1	0	0
CJ75-DY-TE1	0	0.00	1	1	1	1	0	0
CJ75-DY-TE2	0	0.01	0	1	1	1	0	0
CJ75-DY-TE3	3.6	1.37	0	0	0	0	1	0
CJ75-DY-TEO	0.4	0.54	0	0	0	0	1	0
CJ75-DY-TPO(STP)	0.1	0.89	0	0	0	0	1	0
CJ75-DY-PGa/IPa(FSTv)	2.2	2.01	0	0	0	0	1	1
CJ75-DY-A36	0.05	0.08	0	0	1	0	0	0
CJ75-DY-TPPro	0.05	0.00	0	0	1	1	0	0
CJ75-DY-Ent	0.1	0.00	0	0	0	0	0	0
CJ75-DY-TF;TL	0	0.00	1	1	1	1	0	0
CJ75-DY-TH	0.1	0.07	0	0	0	0	0	0
CJ75-DY-TFO;TLO	0.2	0.00	0	0	0	0	0	0
CJ75-DY-AIP	0.4	0.29	0	0	0	0	1	0
CJ75-DY-LIP	1.1	0.14	0	0	0	0	1	0
CJ75-DY-MIP	1.2	0.03	0	0	0	0	0	0
CJ75-DY-VIP	0	0.01	0	1	1	1	0	0
CJ75-DY-PE;PEC	0.4	0.29	0	0	0	0	1	0
CJ75-DY-PF	0	0.00	1	1	1	1	0	0
CJ75-DY-PFG	0.05	0.01	0	0	1	1	0	0
CJ75-DY-PG	0.1	0.07	0	0	0	0	0	0
CJ75-DY-OPt	0.2	0.00	0	0	0	0	0	0
CJ75-DY-PGM	0.05	0.00	0	0	1	1	0	0
CJ75-DY-A31	0.05	0.00	0	0	1	1	0	0
CJ75-DY-A29a-c;A29d	0.1	0.07	0	0	0	0	0	0
CJ75-DY-A30	0.1	0.01	0	0	0	0	0	0
CJ75-DY-A23a	0.05	0.01	0	0	1	1	0	0
CJ75-DY-A23b;A23c	0	0.00	1	1	1	1	0	0
CJ75-DY-A23V	0.3	0.11	0	0	0	0	1	0
CJ75-DY-ProSt	0.05	0.00	0	0	1	1	0	0
CJ75-DY-V2	1.3	10.91	0	0	0	0	1	1
CJ75-DY-V3(VLP)	1.7	5.95	0	0	0	0	1	1
CJ75-DY-V4(VLA)	2.9	3.28	0	0	0	0	1	1
CJ75-DY-V5(MT)	4	1.66	0	0	0	0	1	0
CJ75-DY-V4T(MTC)	4.1	1.25	0	0	0	0	1	0
CJ75-DY-MST	1.3	4.38	0	0	0	0	1	1
CJ75-DY-FST	5.1	5.50	0	0	0	0	1	1
CJ75-DY-V6(DM)	1.7	11.87	0	0	0	0	1	1
CJ75-DY-V3A(DA)	8.5	1.69	0	0	0	0	1	0
CJ75-DY-A19DI	1.7	0.36	0	0	0	0	1	0
CJ75-DY-V6A(PPM)	0	0.03	0	1	1	1	0	0
CJ75-DY-A19M	0.8	0.76	0	0	0	0	1	0
CJ108-FE-A9	0	0.00	1	1	1	1	0	0
CJ108-FE-A10	0.1	0.14	0	0	0	0	1	0
CJ108-FE-A46D;A46V	5.6	4.20	0	0	0	0	1	1
CJ108-FE-A8b	0.5	0.90	0	0	0	0	1	0
CJ108-FE-A8aD	1.8	2.23	0	0	0	0	1	1
CJ108-FE-A8aV	0	0.00	1	1	1	1	0	0
CJ108-FE-A47L(12L)	36.9	40.95	0	0	0	0	1	1
CJ108-FE-A47M(12M)	1.5	2.98	0	0	0	0	1	1
CJ108-FE-A47O(12O)	1.5	0.11	0	0	0	0	1	0
CJ108-FE-A45	6.4	3.45	0	0	0	0	1	1
CJ108-FE-ProM(PrCO)	0.5	0.04	0	0	0	0	0	0
CJ108-FE-A11	0	0.07	0	1	1	0	0	0
CJ108-FE-A13a;A13b	0	0.00	1	1	1	1	0	0
CJ108-FE-A13L	0.1	0.36	0	0	0	0	1	0
CJ108-FE-A13M	0	0.00	1	1	1	1	0	0
CJ108-FE-Gu	0	0.00	1	1	1	1	0	0
CJ108-FE-OPAl	0	0.00	1	1	1	1	0	0
CJ108-FE-OPro	0	0.00	1	1	1	1	0	0
CJ108-FE-A14R;A14C	0	0.00	1	1	1	1	0	0
CJ108-FE-A32	0.05	0.04	0	0	1	1	0	0
CJ108-FE-A32V	0	0.00	1	1	1	1	0	0
CJ108-FE-A4ab;A4c	0	0.00	1	1	1	1	0	0
CJ108-FE-A6DC	0.6	0.11	0	0	0	0	1	0
CJ108-FE-A6DR	2.7	1.01	0	0	0	0	1	0
CJ108-FE-A6M	0.05	0.11	0	0	1	0	0	0

CJ108-FE-A6Va;A6Vb	3.2	3.59	0	0	0	0	1	1
CJ108-FE-A8C	4.7	5.75	0	0	0	0	1	1
CJ108-FE-A24b	0	0.00	1	1	1	1	0	0
CJ108-FE-A24c;A24d	0	0.00	1	1	1	1	0	0
CJ108-FE-A3a	0	0.00	1	1	1	1	0	0
CJ108-FE-A1/2	0.1	0.43	0	0	0	0	1	0
CJ108-FE-S2E;S2I;S2PV;S2PR	0.2	0.14	0	0	0	0	1	0
CJ108-FE-AI;DI;GI;IPro;PaIL;PaIM;ReI;TPro	0.05	0.07	0	0	1	0	0	0
CJ108-FE-AuA1;AuAL;AuCL;AuCM;AuML;AuR;AuRM;AuRTL;AuRTM;AuRT	0.05	0.04	0	0	1	1	0	0
CJ108-FE-AuRPB;AuCPB	0	0.04	0	1	1	1	0	0
CJ108-FE-TPt	0.05	0.22	0	0	1	0	0	0
CJ108-FE-STR	0	0.00	1	1	1	1	0	0
CJ108-FE-TE1	0	0.00	1	1	1	1	0	0
CJ108-FE-TE2	0	0.00	1	1	1	1	0	0
CJ108-FE-TE3	0.1	0.07	0	0	0	0	0	0
CJ108-FE-TEO	0	0.00	1	1	1	1	0	0
CJ108-FE-TPO(STP)	0	0.43	0	1	1	0	0	0
CJ108-FE-PGa/IPa(FSTv)	0.4	0.07	0	0	0	0	0	0
CJ108-FE-A36	0	0.00	1	1	1	1	0	0
CJ108-FE-TPPro	0	0.00	1	1	1	1	0	0
CJ108-FE-Ent	0	0.00	1	1	1	1	0	0
CJ108-FE-TF;TL	0	0.00	1	1	1	1	0	0
CJ108-FE-TH	0.1	0.11	0	0	0	0	1	0
CJ108-FE-TFO;TLO	0	0.00	1	1	1	1	0	0
CJ108-FE-AIP	2	0.22	0	0	0	0	1	0
CJ108-FE-LIP	0.5	0.14	0	0	0	0	1	0
CJ108-FE-MIP	1.5	1.69	0	0	0	0	1	0
CJ108-FE-VIP	1.1	0.25	0	0	0	0	1	0
CJ108-FE-PE;PEC	5.1	6.07	0	0	0	0	1	1
CJ108-FE-PF	2	1.44	0	0	0	0	1	0
CJ108-FE-PFG	2.8	1.90	0	0	0	0	1	0
CJ108-FE-PG	3.3	2.05	0	0	0	0	1	1
CJ108-FE-OPt	1.6	2.55	0	0	0	0	1	1
CJ108-FE-PGM	1.3	2.23	0	0	0	0	1	1
CJ108-FE-A31	1.7	0.40	0	0	0	0	1	0
CJ108-FE-A29a-c;A29d	0.05	0.04	0	0	1	1	0	0
CJ108-FE-A30	0	0.00	1	1	1	1	0	0
CJ108-FE-A23a	0.1	0.07	0	0	0	0	0	0
CJ108-FE-A23b;A23c	1.7	0.68	0	0	0	0	1	0
CJ108-FE-A23V	0.2	0.00	0	0	0	0	0	0
CJ108-FE-ProSt	0	0.00	1	1	1	1	0	0
CJ108-FE-V2	0.6	0.65	0	0	0	0	1	0
CJ108-FE-V3(VLP)	0.3	0.79	0	0	0	0	1	0
CJ108-FE-V4(VLA)	0.2	0.18	0	0	0	0	1	0
CJ108-FE-V5(MT)	1.4	1.47	0	0	0	0	1	0
CJ108-FE-V4T(MTC)	0.1	0.14	0	0	0	0	1	0
CJ108-FE-MST	2.6	1.83	0	0	0	0	1	0
CJ108-FE-FST	0.5	0.36	0	0	0	0	1	0
CJ108-FE-V6(DM)	0.1	1.47	0	0	0	0	1	0
CJ108-FE-V3A(DA)	0.4	0.61	0	0	0	0	1	0
CJ108-FE-A19DI	0	0.00	1	1	1	1	0	0
CJ108-FE-V6A(PPM)	0.7	2.66	0	0	0	0	1	1
CJ108-FE-A19M	1.2	2.41	0	0	0	0	1	1
CJ94-DY-A9	0.9	0.05	0	0	0	0	0	0
CJ94-DY-A10	0.9	0.56	0	0	0	0	1	0
CJ94-DY-A46D;A46V	5.5	3.34	0	0	0	0	1	1
CJ94-DY-A8b	0.1	1.28	0	0	0	0	1	0
CJ94-DY-A8aD	0.7	2.29	0	0	0	0	1	1
CJ94-DY-A8aV	0	0.00	1	1	1	1	0	0
CJ94-DY-A47L(12L)	28.6	21.64	0	0	0	0	1	1
CJ94-DY-A47M(12M)	0.9	5.12	0	0	0	0	1	1
CJ94-DY-A47O(12O)	0.2	0.29	0	0	0	0	1	0
CJ94-DY-A45	8	13.22	0	0	0	0	1	1
CJ94-DY-ProM(PrCO)	0.05	0.06	0	0	1	0	0	0
CJ94-DY-A11	0	0.04	0	1	1	1	0	0
CJ94-DY-A13a;A13b	0.05	0.00	0	0	1	1	0	0
CJ94-DY-A13L	0.4	1.14	0	0	0	0	1	0
CJ94-DY-A13M	0.3	0.15	0	0	0	0	1	0
CJ94-DY-Gu	0.05	0.02	0	0	1	1	0	0
CJ94-DY-OPAI	0	0.00	1	1	1	1	0	0
CJ94-DY-OPro	0	0.02	0	1	1	1	0	0
CJ94-DY-A14R;A14C	0	0.00	1	1	1	1	0	0
CJ94-DY-A32	0	0.00	1	1	1	1	0	0
CJ94-DY-A32V	0	0.00	1	1	1	1	0	0
CJ94-DY-A4ab;A4c	2.6	0.02	0	0	0	0	0	0
CJ94-DY-A6DC	3.3	3.13	0	0	0	0	1	1
CJ94-DY-A6DR	8.7	4.55	0	0	0	0	1	1
CJ94-DY-A6M	0.1	0.23	0	0	0	0	1	0
CJ94-DY-A6Va;A6Vb	6.3	6.54	0	0	0	0	1	1
CJ94-DY-A8C	1.7	4.55	0	0	0	0	1	1
CJ94-DY-A24b	0.1	0.10	0	0	0	0	1	0
CJ94-DY-A24c;A24d	0	0.05	0	1	1	1	0	0
CJ94-DY-A3a	0.1	0.12	0	0	0	0	1	0
CJ94-DY-A1/2	0.1	0.05	0	0	0	0	0	0
CJ94-DY-S2E;S2I;S2PV;S2PR	0.05	0.00	0	0	1	1	0	0
CJ94-DY-AI;DI;GI;IPro;PaIL;PaIM;ReI;TPro	0.1	0.00	0	0	0	0	0	0
CJ94-DY-AuA1;AuAL;AuCL;AuCM;AuML;AuR;AuRM;AuRTL;AuRTM;AuRT	0.05	0.12	0	0	1	0	0	0
CJ94-DY-AuRPB;AuCPB	0.05	0.02	0	0	1	1	0	0
CJ94-DY-TPt	0.05	0.16	0	0	1	0	0	0
CJ94-DY-STR	0	0.00	1	1	1	1	0	0
CJ94-DY-TE1	0.7	0.01	0	0	0	0	0	0
CJ94-DY-TE2	0.4	0.02	0	0	0	0	0	0
CJ94-DY-TE3	2.7	2.62	0	0	0	0	1	1
CJ94-DY-TEO	0.8	0.14	0	0	0	0	1	0
CJ94-DY-TPO(STP)	0.05	0.38	0	0	1	0	0	0
CJ94-DY-PGa/IPa(FSTv)	1.7	1.40	0	0	0	0	1	0
CJ94-DY-A36	0	0.00	1	1	1	1	0	0
CJ94-DY-TPPro	0	0.00	1	1	1	1	0	0
CJ94-DY-Ent	0	0.00	1	1	1	1	0	0
CJ94-DY-TF;TL	0	0.00	1	1	1	1	0	0
CJ94-DY-TH	0	0.00	1	1	1	1	0	0
CJ94-DY-TFO;TLO	0.05	0.00	0	0	1	1	0	0
CJ94-DY-AIP	0	1.48	0	1	1	0	0	0
CJ94-DY-LIP	3.7	1.72	0	0	0	0	1	0
CJ94-DY-MIP	1.9	2.01	0	0	0	0	1	1
CJ94-DY-VIP	0	1.28	0	1	1	0	0	0
CJ94-DY-PE;PEC	1.8	2.00	0	0	0	0	1	1
CJ94-DY-PF	0.4	0.00	0	0	0	0	0	0
CJ94-DY-PFG	1.2	0.28	0	0	0	0	1	0
CJ94-DY-PG	0.2	0.50	0	0	0	0	1	0
CJ94-DY-OPt	0.9	2.13	0	0	0	0	1	1
CJ94-DY-PGM	0.2	0.62	0	0	0	0	1	0

CJ94-DY-A31	0.1	0.01	0	0	0	0	0	0
CJ94-DY-A29a-c;A29d	0.3	0.60	0	0	0	0	1	0
CJ94-DY-A30	0.1	0.08	0	0	0	0	0	0
CJ94-DY-A23a	0.05	0.02	0	0	1	1	0	0
CJ94-DY-A23b;A23c	0.2	0.00	0	0	0	0	0	0
CJ94-DY-A23V	0.2	0.00	0	0	0	0	0	0
CJ94-DY-ProSt	0	0.00	1	1	1	1	0	0
CJ94-DY-V2	0.2	0.39	0	0	0	0	1	0
CJ94-DY-V3(VLP)	0.2	0.80	0	0	0	0	1	0
CJ94-DY-V4(VLA)	2.3	0.74	0	0	0	0	1	0
CJ94-DY-V5(MT)	0.6	1.69	0	0	0	0	1	0
CJ94-DY-V4T(MTC)	3	3.52	0	0	0	0	1	1
CJ94-DY-MST	1.3	1.39	0	0	0	0	1	0
CJ94-DY-FST	1.8	2.11	0	0	0	0	1	1
CJ94-DY-V6(DM)	0.1	1.35	0	0	0	0	1	0
CJ94-DY-V3A(DA)	2.4	0.73	0	0	0	0	1	0
CJ94-DY-A19DI	0.05	0.33	0	0	1	0	0	0
CJ94-DY-V6A(PPM)	0	0.41	0	1	1	0	0	0
CJ94-DY-A19M	0.2	0.18	0	0	0	0	1	0
