# Supplementary material for: Towards a comprehensive atlas of cortical connections in a primate brain: Mapping tracer injection studies of the common marmoset into a reference digital template
Source: J Comp Neurol. 2016 Jun 3;524(11):2161–81. doi: 10.1002/cne.24023 (PMC4892968; doi:10.1002/cne.24023)
Supplement: Supplementary file 3 — Supporting Information [file CNE-524-2161-s003.pdf]

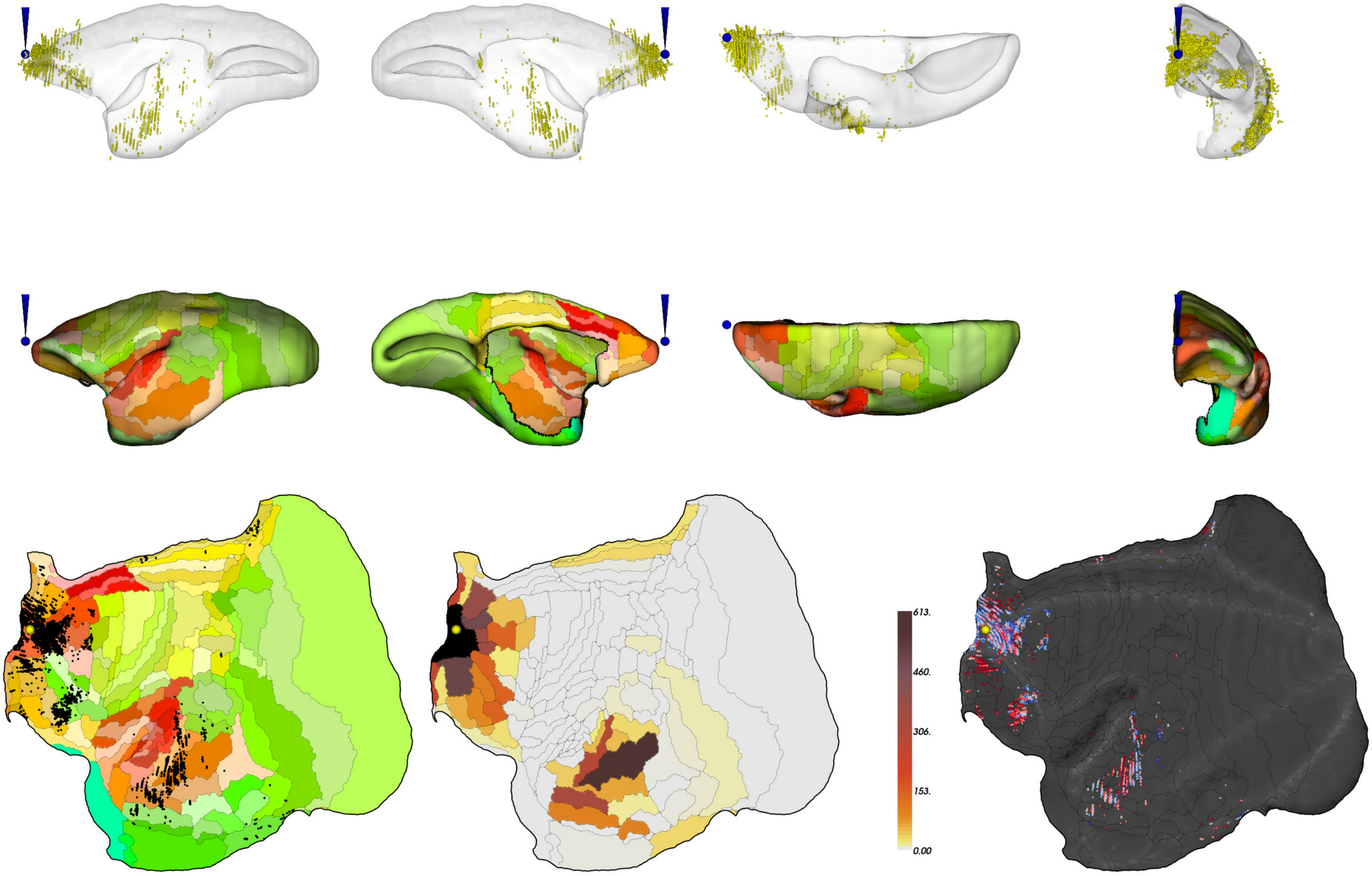

Case: CJ70 DY

Structure: A10

A-P: -19.5 mm, M-L: 1.0 mm, D-V: 11.9 mm

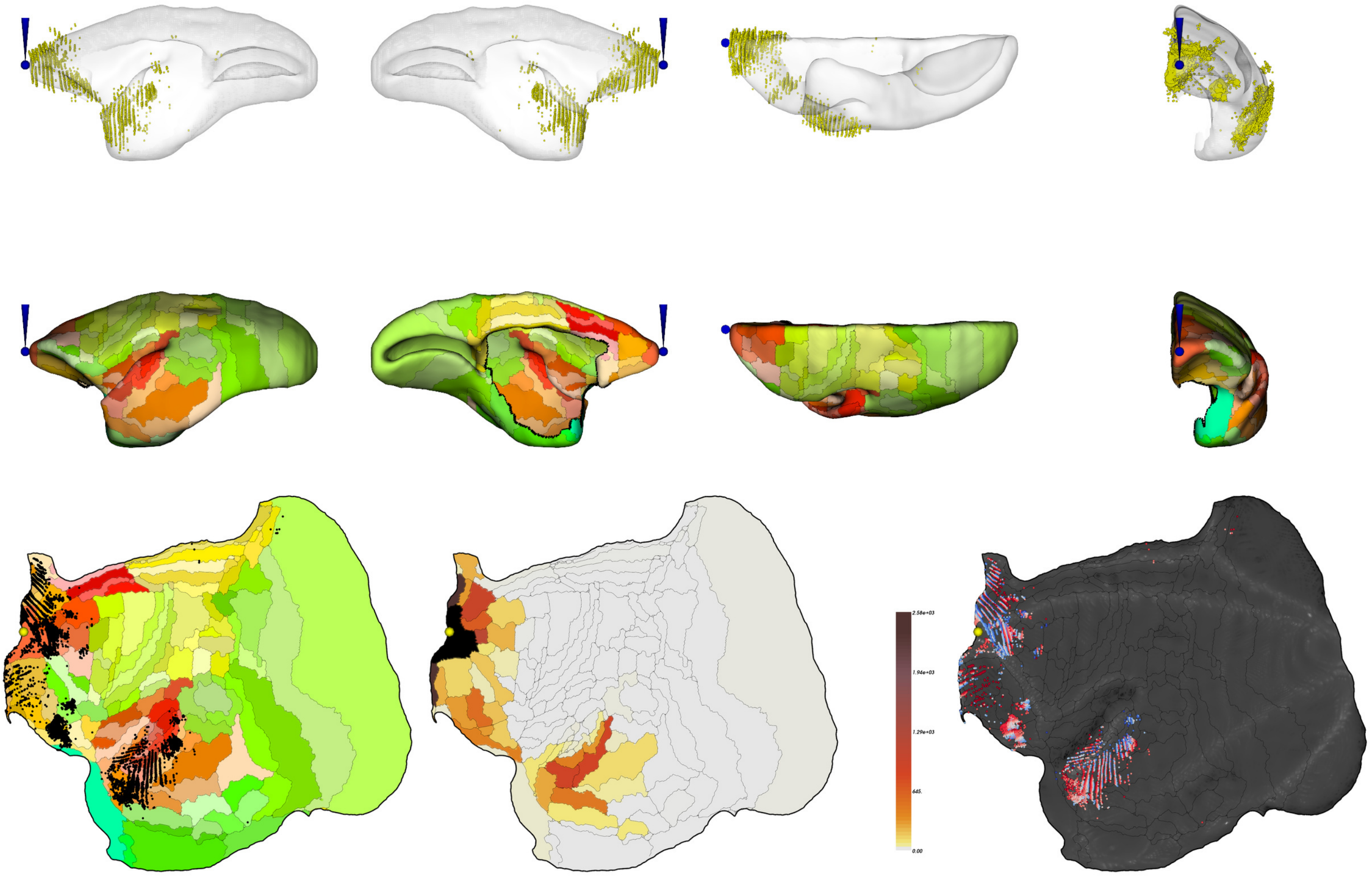

Case: CJ71 DY

Structure: A10

A-P: -19.5 mm, M-L: 1.2 mm, D-V: 11.1 mm

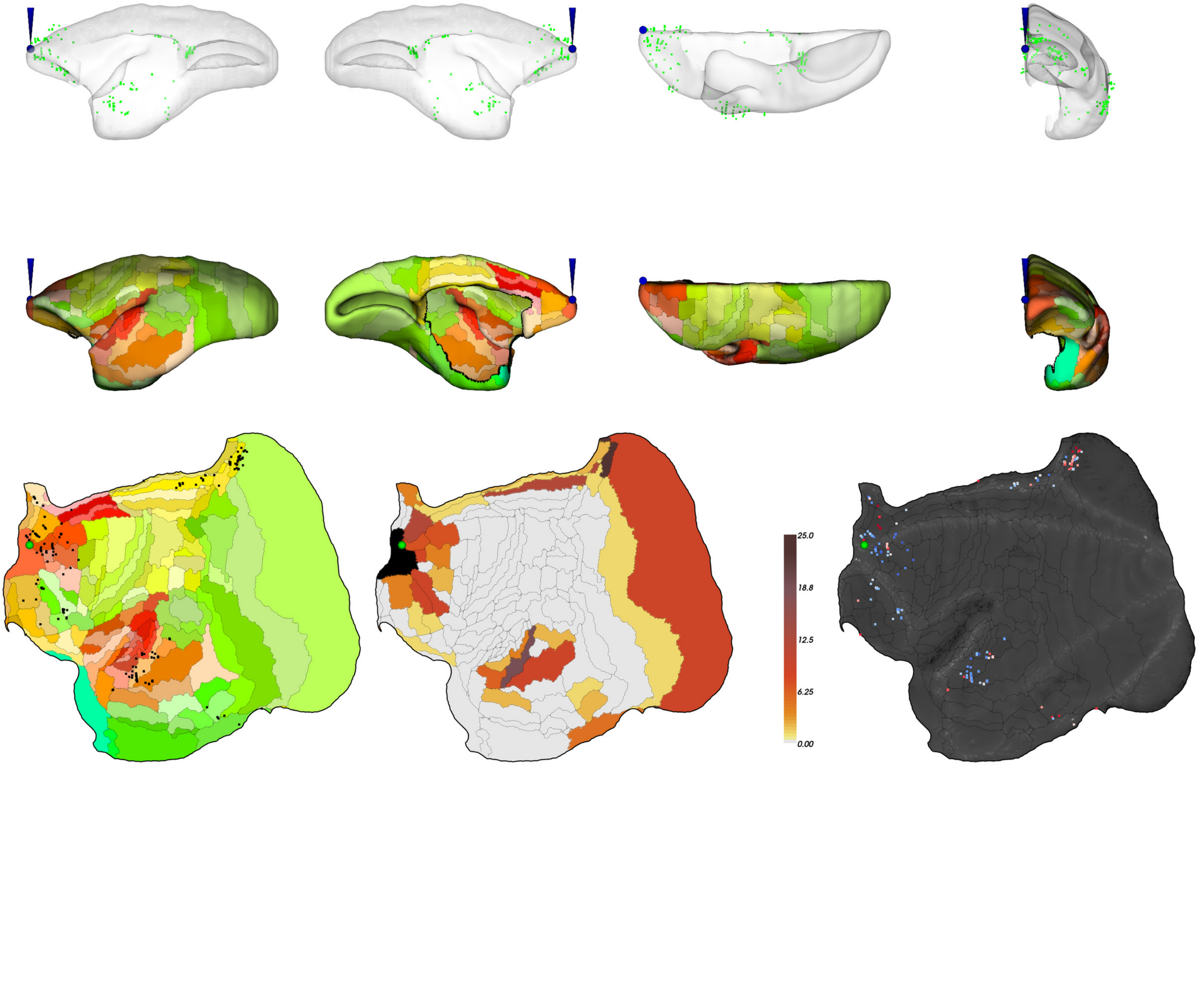

Case: CJ71 FE

Structure: A10

A-P: -18.5 mm, M-L: 0.5 mm, D-V: 12.1 mm

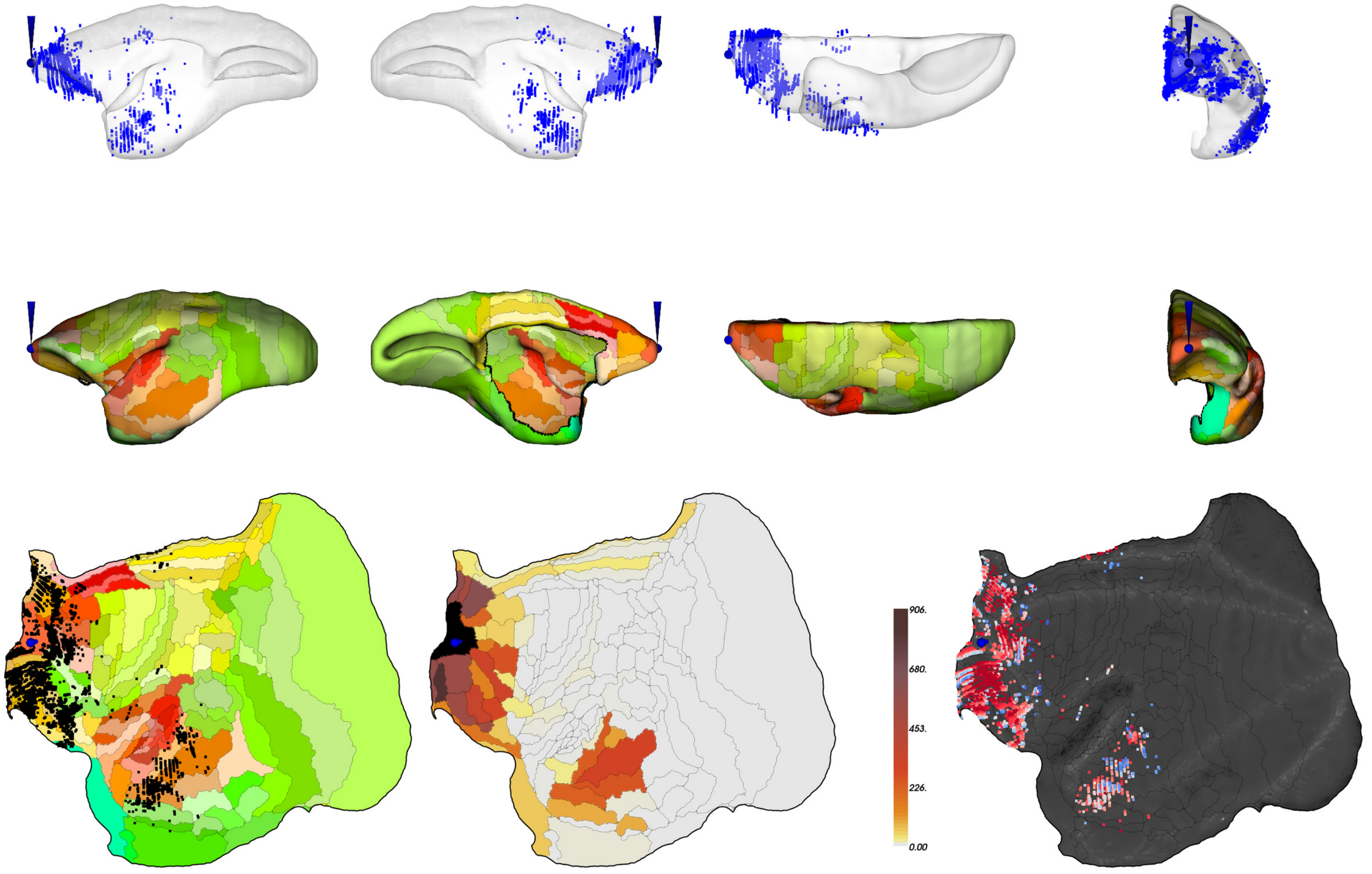

Case: CJ71 FB

Structure: A10

A-P: -19.0 mm, M-L: 3.0 mm, D-V: 11.5 mm

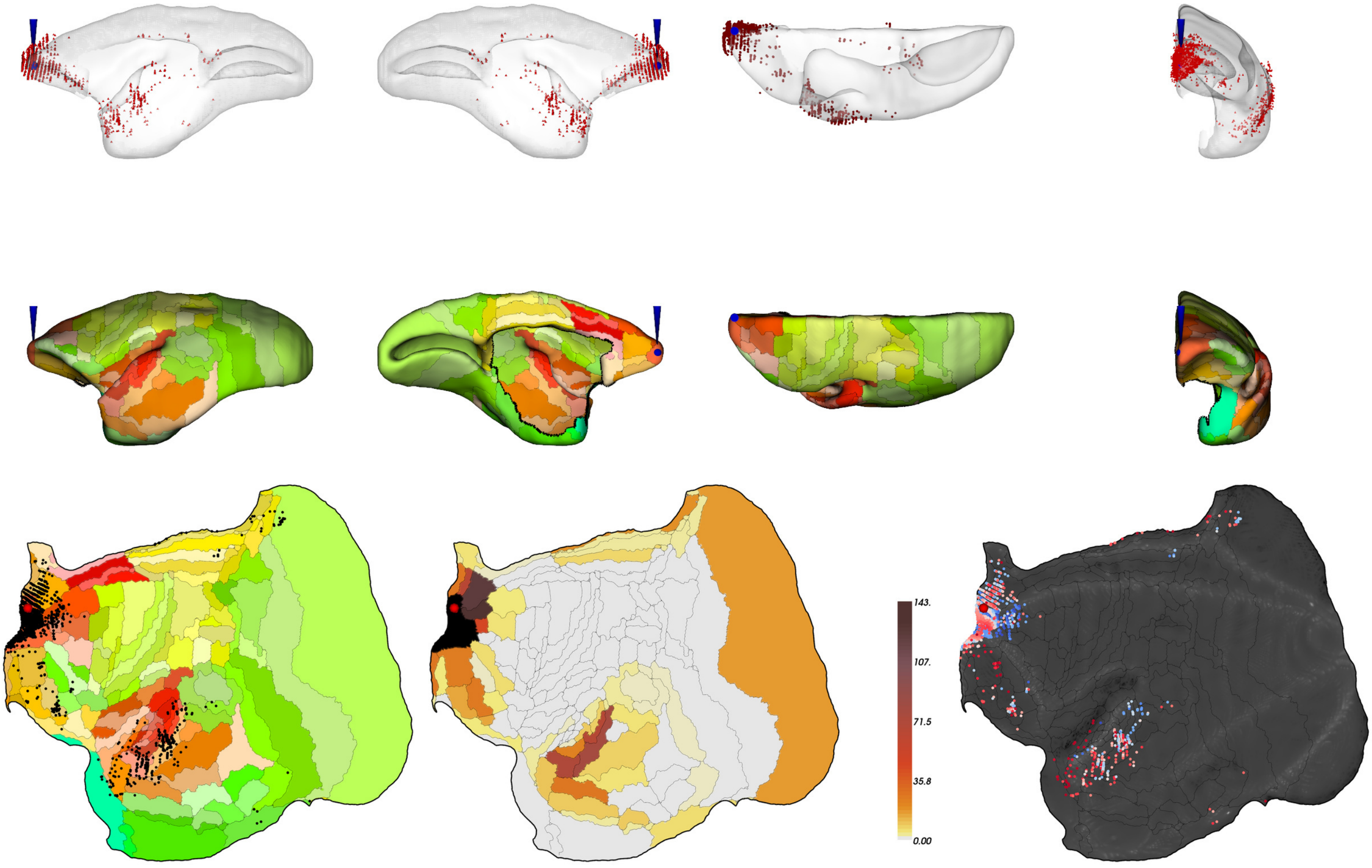

Case: CJ73 FR

Structure: A10

A-P: -18.5 mm, M-L: 0.7 mm, D-V: 11.1 mm

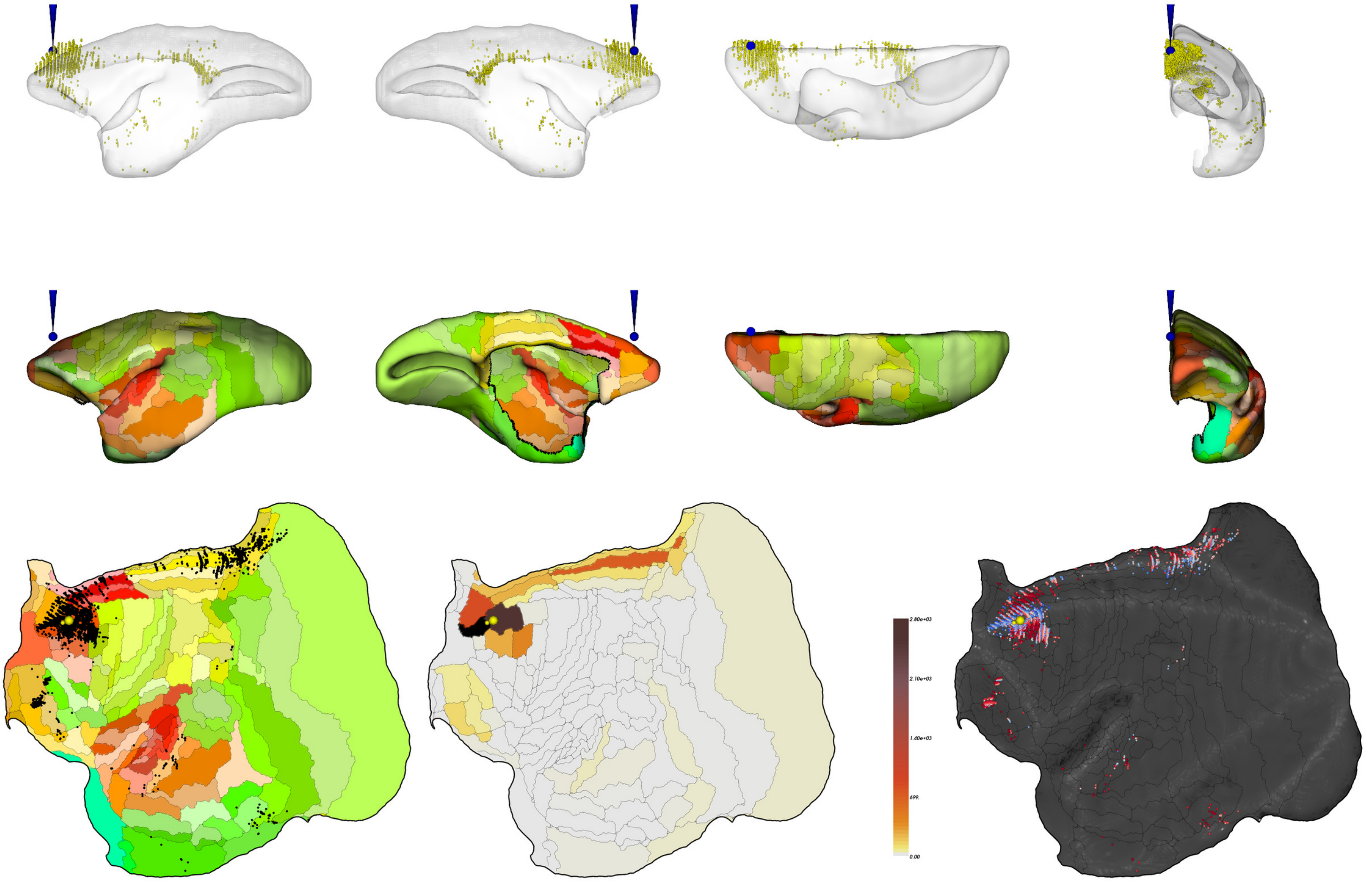

Case: CJ73 DY

Structure: A9

A-P: -16.5 mm, M-L: 1.0 mm, D-V: 13.6 mm

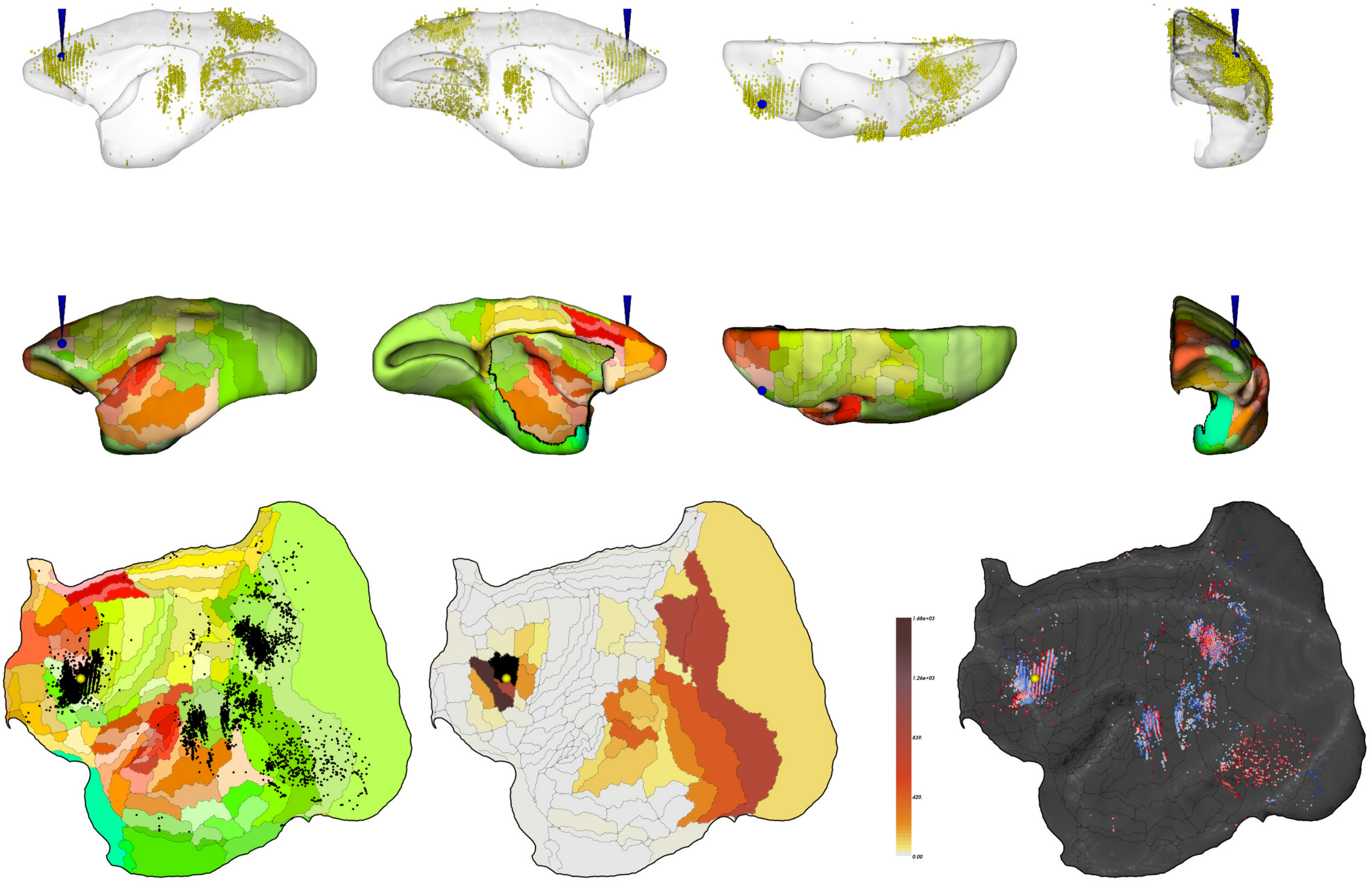

Case: CJ75 DY

Structure: A8aV

A-P: -15.0 mm, M-L: 7.0 mm, D-V: 12.5 mm

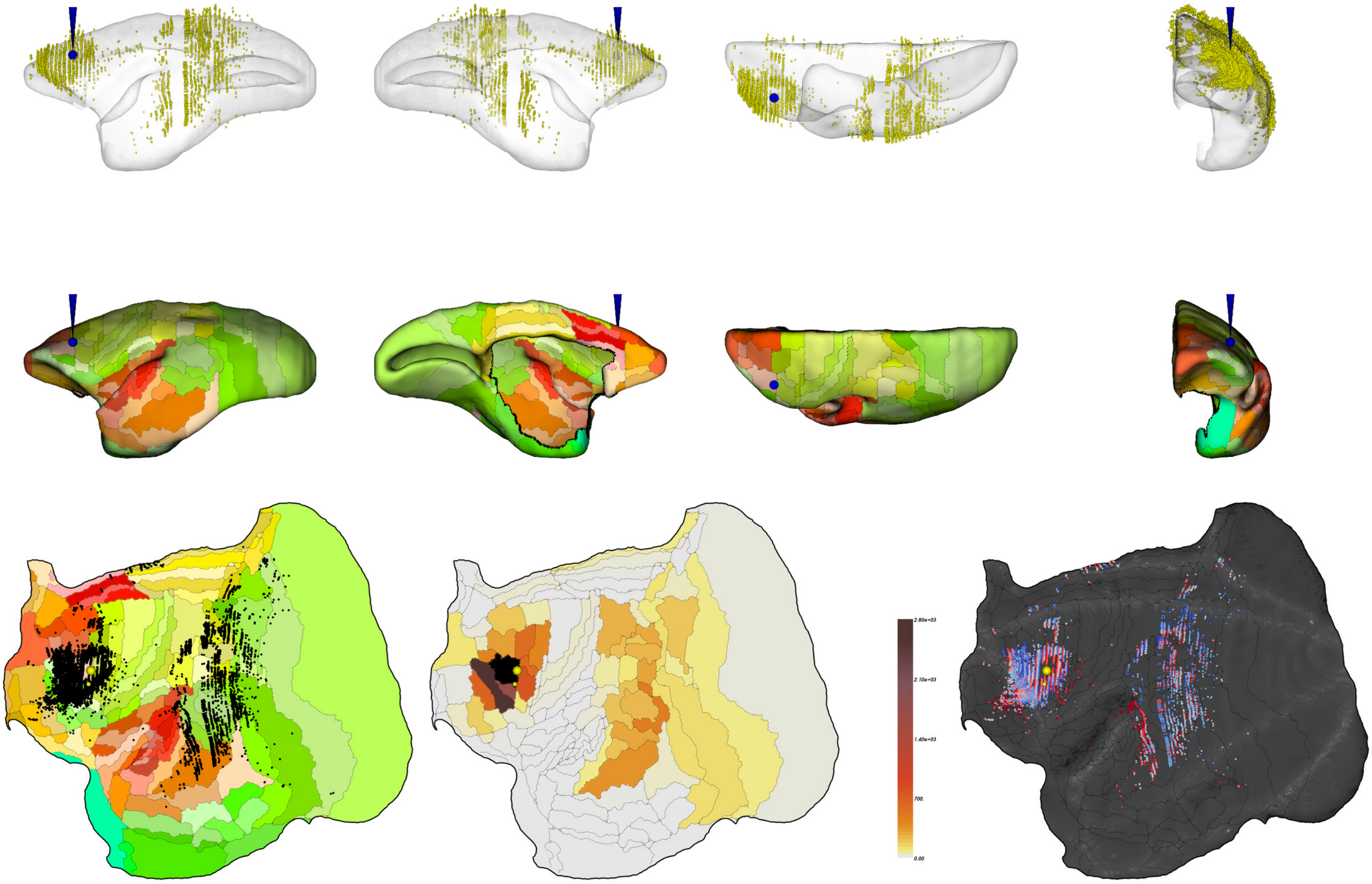

Case: CJ94 DY

Structure: A8aV

A-P: -14.0 mm, M-L: 7.4 mm, D-V: 11.8 mm

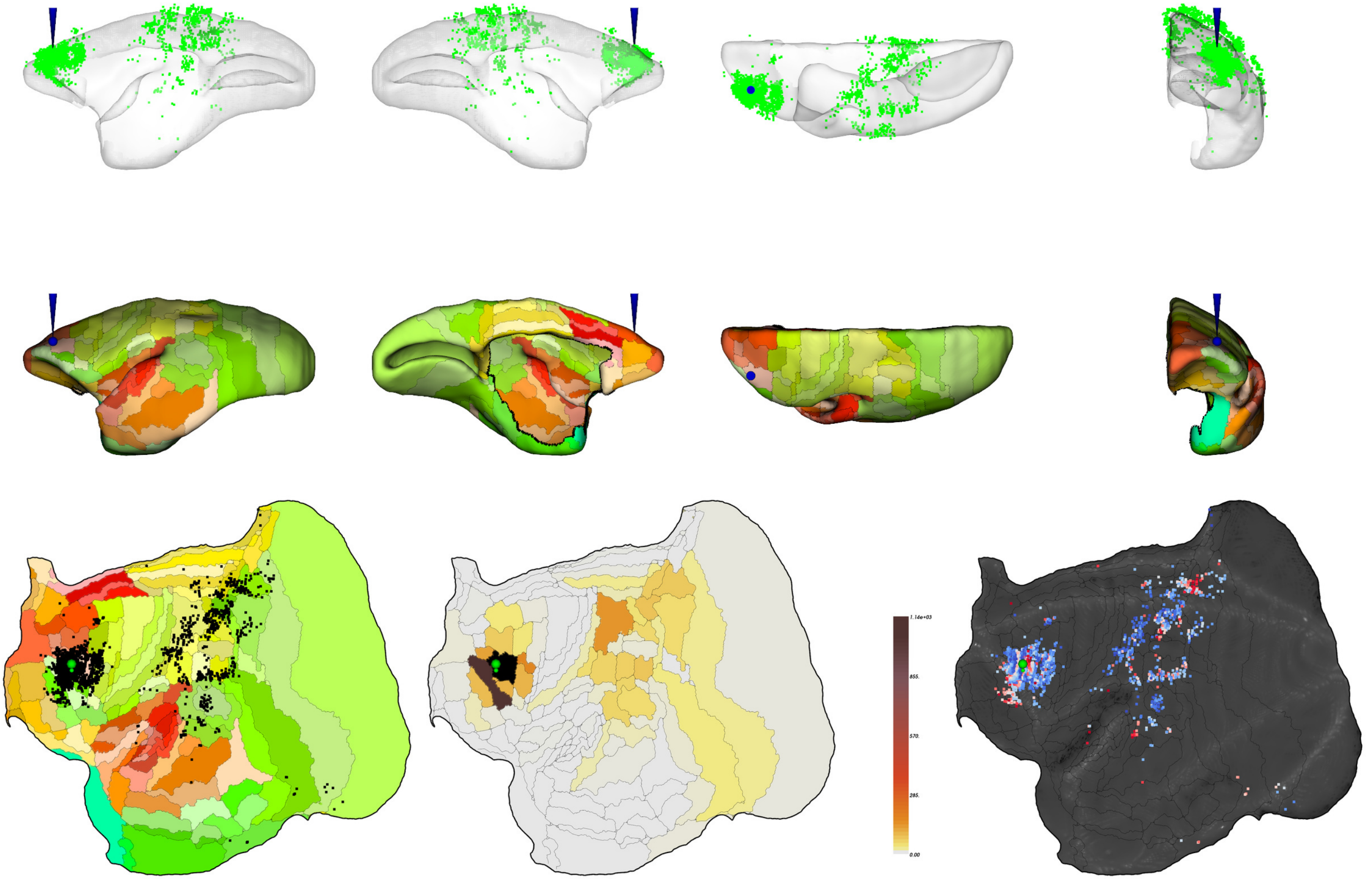

Case: CJ108 FE

Structure: A8aV

A-P: -16.0 mm, M-L: 6.4 mm, D-V: 12.5 mm

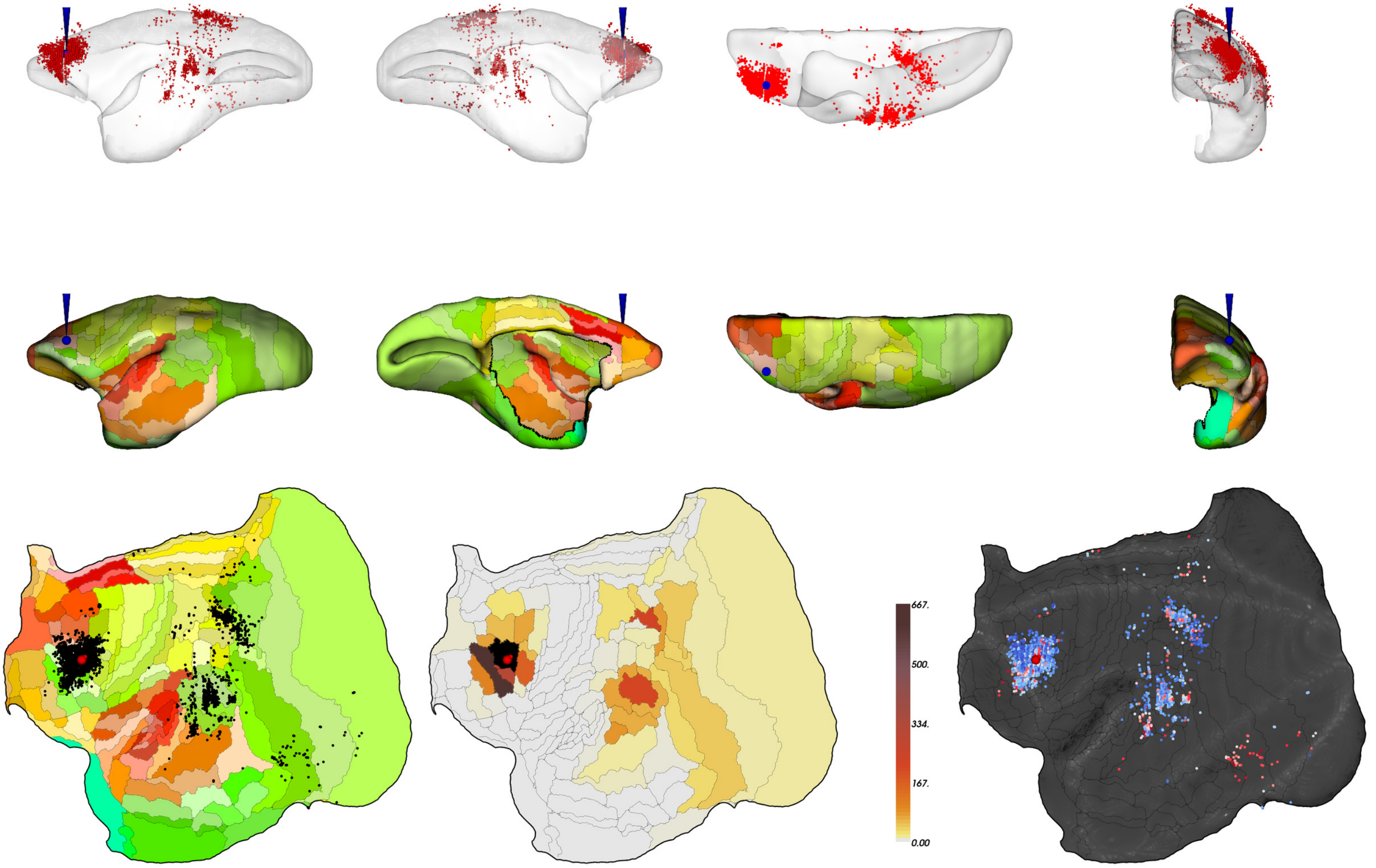

Case: CJ125 FR

Structure: A8aV

A-P: -14.5 mm, M-L: 6.5 mm, D-V: 12.6 mm

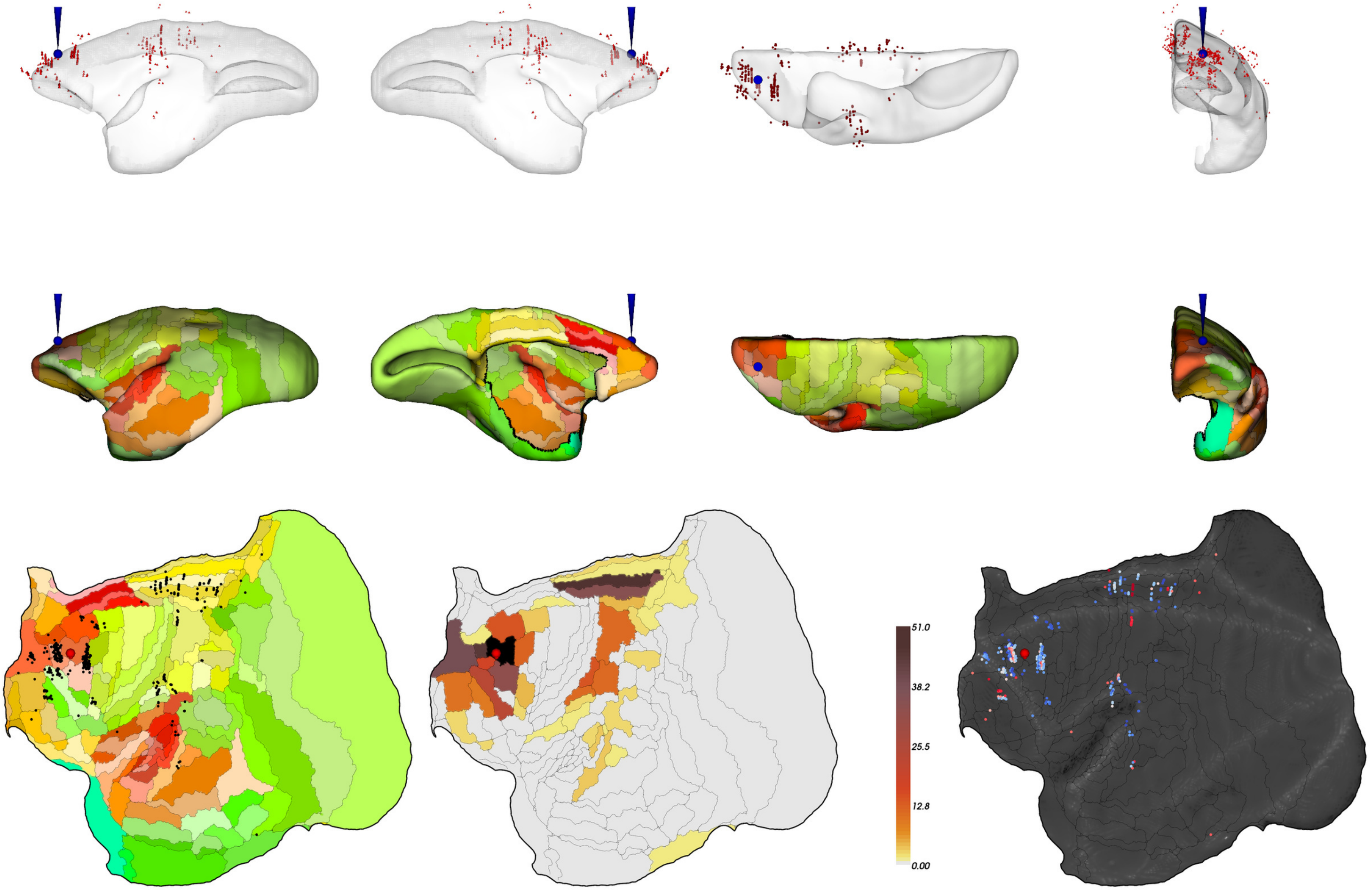

Case: CJ70 FR

Structure: A8aD

A-P: -16.0 mm, M-L: 4.0 mm, D-V: 13.0 mm

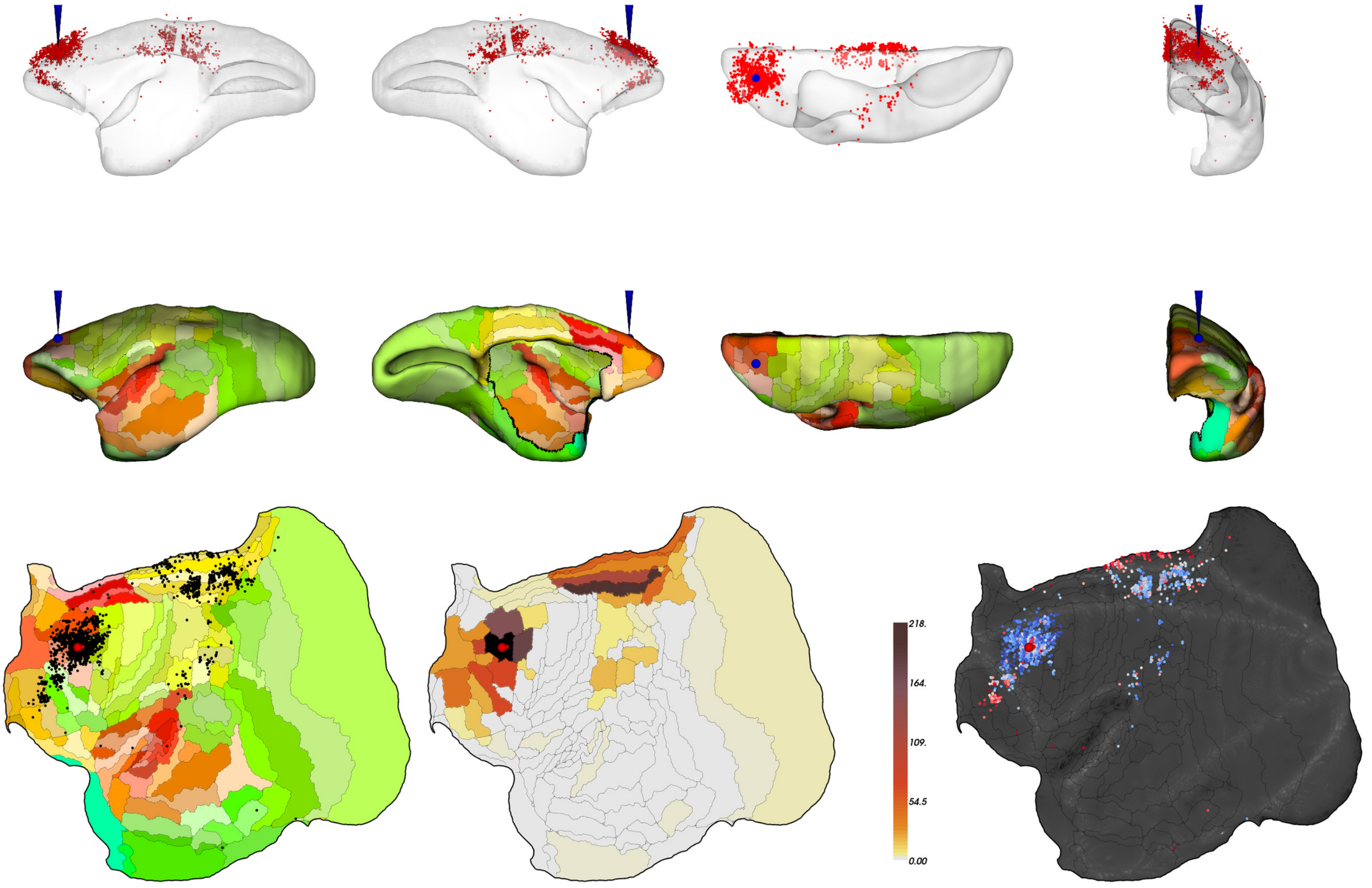

Case: CJ108 FR

Structure: A8aD

A-P: -15.0 mm, M-L: 4.0 mm, D-V: 14.0 mm

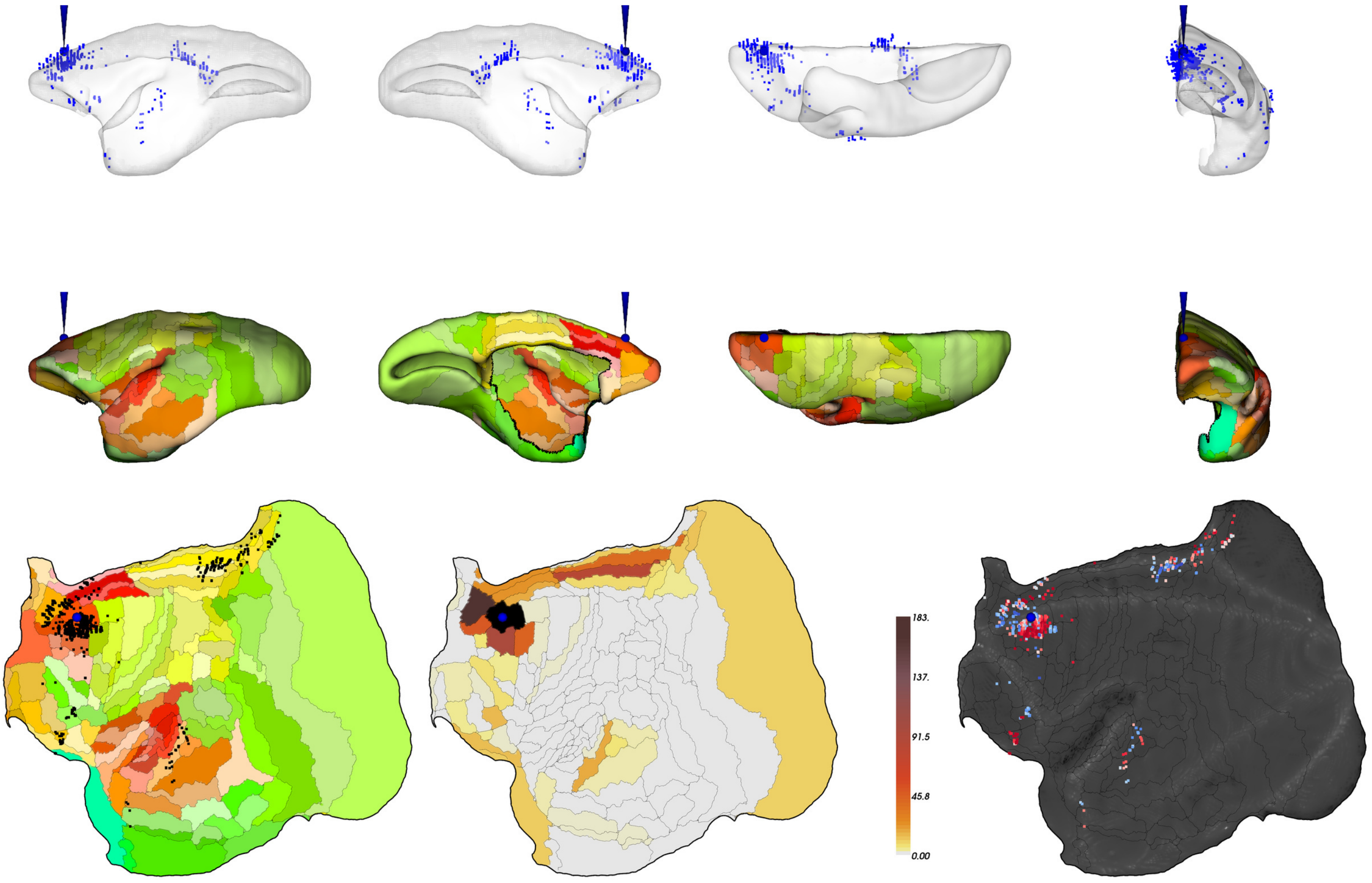

Case: CJ74 FB

Structure: A8b

A-P: -15.0 mm, M-L: 1.5 mm, D-V: 14.1 mm

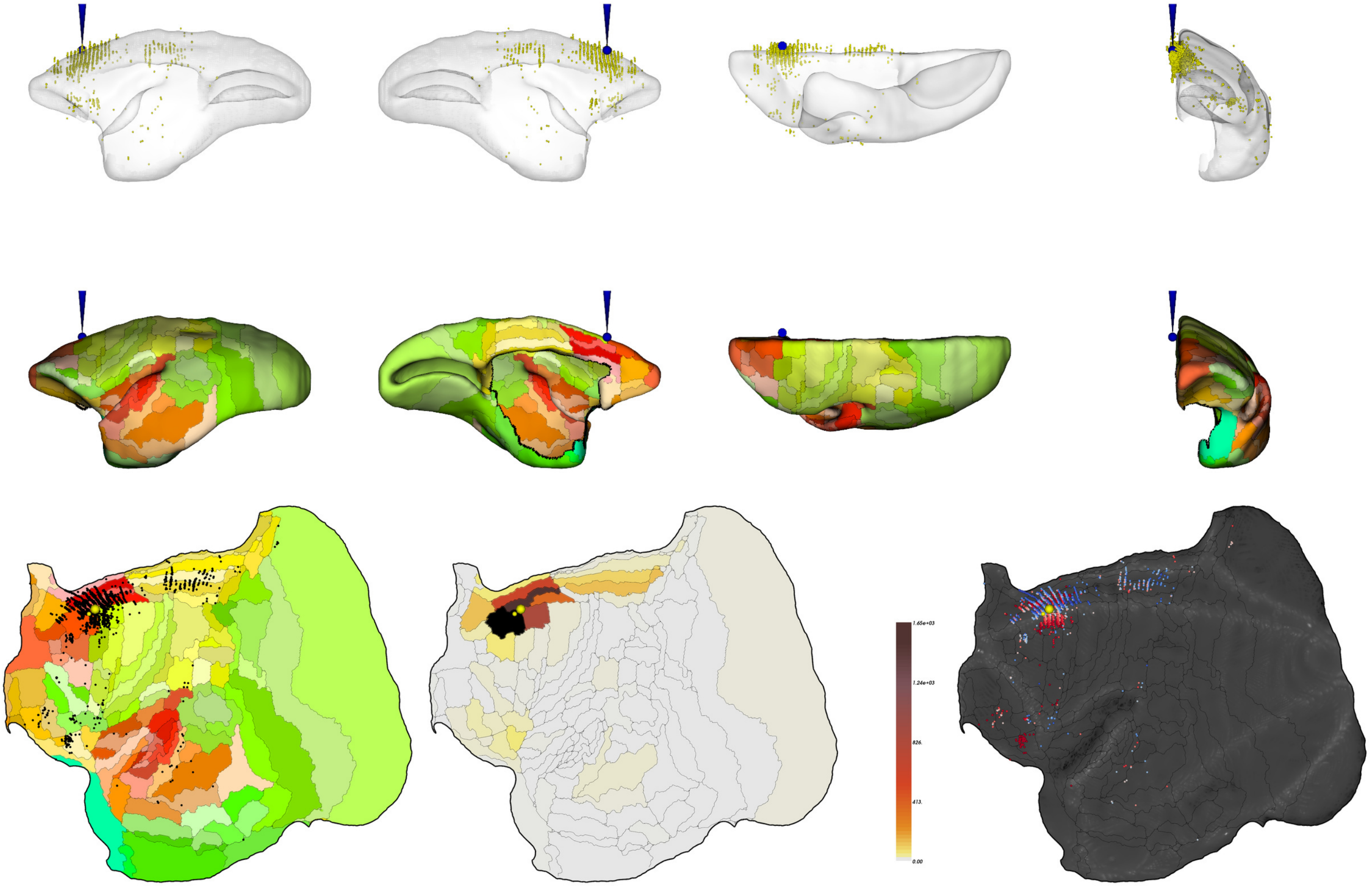

Case: CJ74 DY

Structure: A8b

A-P: -14.0 mm, M-L: 0.5 mm, D-V: 15.0 mm

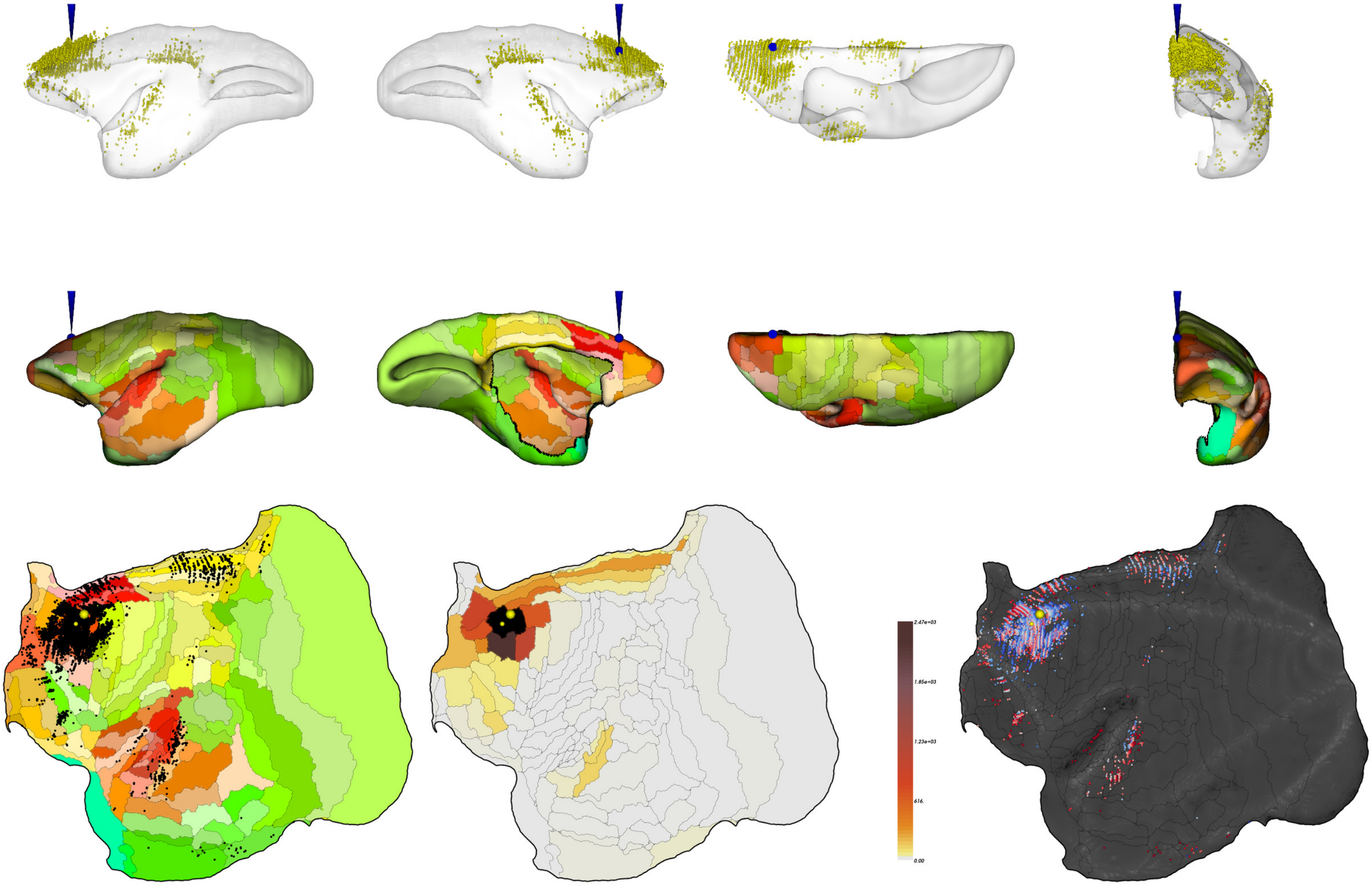

Case: CJ83 DY

Structure: A8b

A-P: -15.5 mm, M-L: 1.4 mm, D-V: 14.3 mm

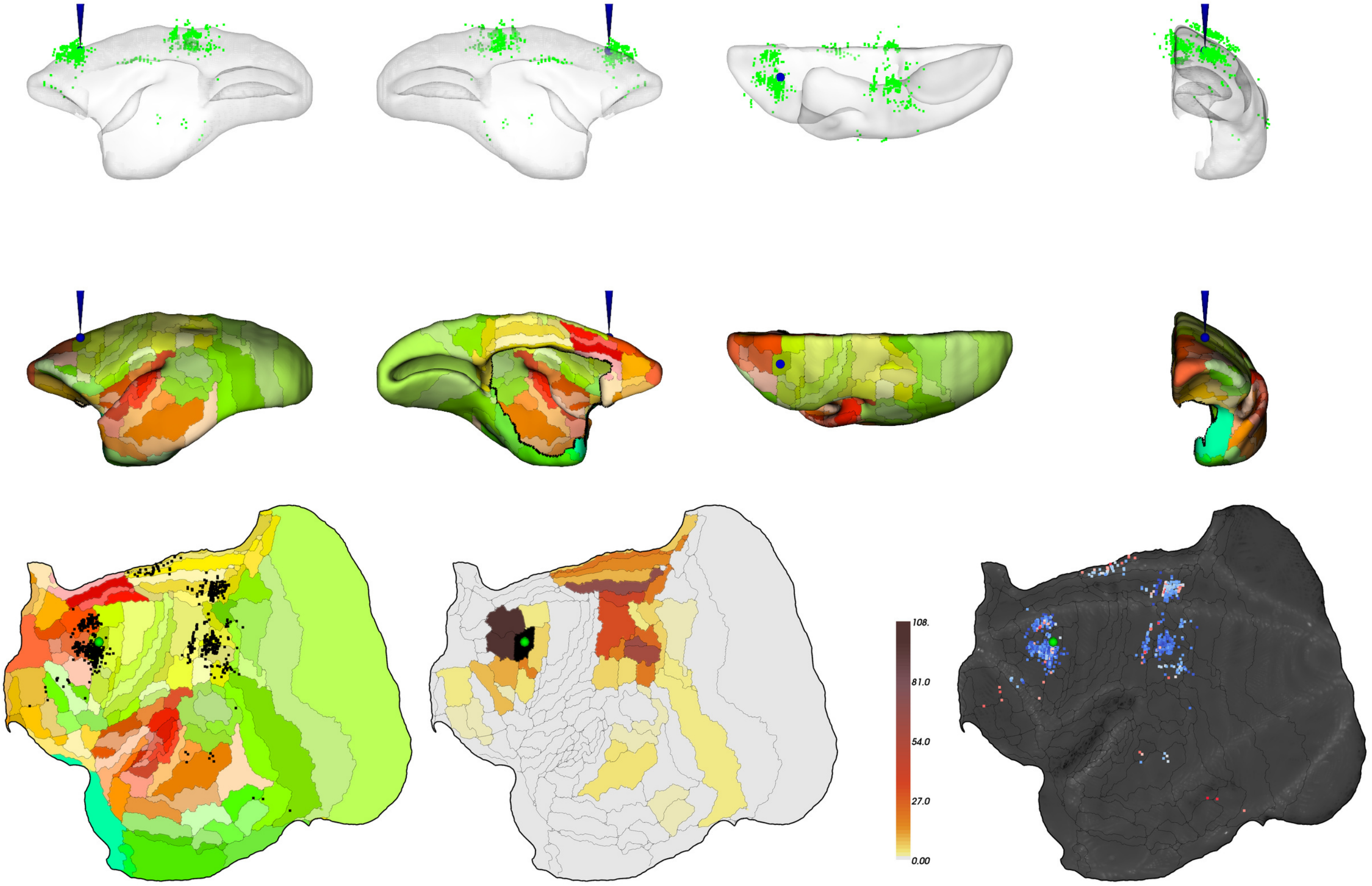

Case: CJ125 FE

Structure: A6DR

A-P: -13.5 mm, M-L: 4.0 mm, D-V: 15.0 mm

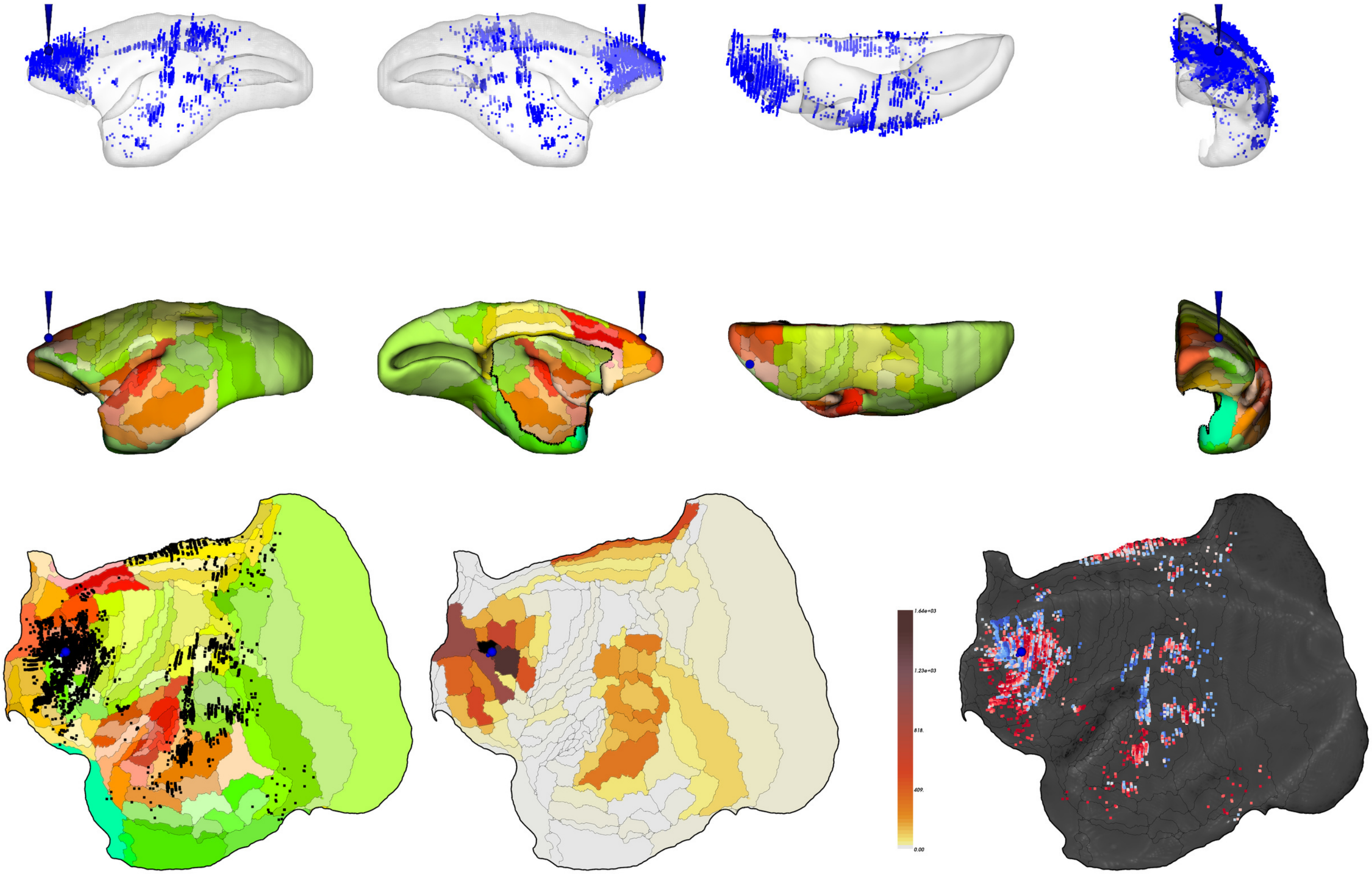

Case: CJ73 FB

Structure: A46V

A-P: -17.0 mm, M-L: 5.1 mm, D-V: 12.5 mm
