# Supplementary material for: Towards a comprehensive atlas of cortical connections in a primate brain: Mapping tracer injection studies of the common marmoset into a reference digital template
Source: J Comp Neurol. 2016 Jun 3;524(11):2161–81. doi: 10.1002/cne.24023 (PMC4892968; doi:10.1002/cne.24023)
Supplement: Supplementary file 4 — Supporting Information [file CNE-524-2161-s004.docx]

S4_marmoset_brain_template.zip

This is a place holder. The current manuscript submission web site in manuscriptcentral does not allow uploading of files in the formats needed for direct access of the 3-D template.

A folder containing the template and instructions will be provided separately to the editor, to be shared with the reviewer. If the paper is accepted, it will be available for free download.
